# Pirfenidone increases IL-10 and improves acute pancreatitis in multiple clinically relevant murine models

**DOI:** 10.1172/jci.insight.141108

**Published:** 2022-01-25

**Authors:** Ejas Palathingal Bava, John George, Mohammad Tarique, Srikanth Iyer, Preeti Sahay, Beatriz Gomez Aguilar, Dujon B. Edwards, Bhuwan Giri, Vrishketan Sethi, Tejeshwar Jain, Prateek Sharma, Utpreksha Vaish, Harrys K. C. Jacob, Anthony Ferrantella, Craig L. Maynard, Ashok K. Saluja, Rajinder K. Dawra, Vikas Dudeja

**Affiliations:** 1 Department of Surgery, University of Alabama at Birmingham, Birmingham, Alabama, USA.; 2 Department of Surgery and Sylvester Comprehensive Cancer Center, University of Miami, Miami, Florida, USA.; 3 Department of Pathology, University of Alabama at Birmingham, Birmingham, Alabama, USA.; 4 Birmingham Veteran Affairs Medical Center, Birmingham, Alabama, USA.

**Keywords:** Gastroenterology, Inflammation, Chemokines, Cytokines, Macrophages

## Abstract

Despite decades of research, there is no specific therapy for acute pancreatitis (AP). In the current study, we have evaluated the efficacy of pirfenidone, an antiinflammatory and antifibrotic agent that is approved by the FDA for treatment of idiopathic pulmonary fibrosis (IPF), in ameliorating local and systemic injury in AP. Our results suggest that treatment with pirfenidone in therapeutic settings (e.g., after initiation of injury), even when administered at the peak of injury, reduces severity of local and systemic injury and inflammation in multiple models of AP. In vitro evaluation suggests that pirfenidone decreases cytokine release from acini and macrophages and disrupts acinar-macrophage crosstalk. Therapeutic pirfenidone treatment increases IL-10 secretion from macrophages preceding changes in histology and modulates the immune phenotype of inflammatory cells with decreased levels of inflammatory cytokines. Antibody-mediated IL-10 depletion, use of IL-10–KO mice, and macrophage depletion experiments confirmed the role of IL-10 and macrophages in its mechanism of action, as pirfenidone was unable to reduce severity of AP in these scenarios. Since pirfenidone is FDA approved for IPF, a trial evaluating the efficacy of pirfenidone in patients with moderate to severe AP can be initiated expeditiously.

## Introduction

Acute pancreatitis (AP), a common gastrointestinal (GI) disorder, is one of the most common causes of hospitalization due to a GI disease in the United States (1). Despite decades of research, there is no specific treatment for AP, and its management continues to be focused on supportive measures (2, 3). Pathogenesis of AP can be broadly divided into 2 phases: an early intraacinar initiation phase and a later phase of local and systemic inflammation. By the time patients with AP present to the hospital, the intraacinar events have already transpired, and the disease has typically advanced to the stage of local and systemic inflammation. As a result, targeting early intraacinar events — for instance, protease activation (4, 5) — has not resulted in improved outcomes in AP. Thus, apart from select few clinical settings, where it may be feasible to target intraacinar events for therapeutic benefit (for instance, prevention of ERCP induced AP and prevention of recurrent AP), specific treatment for AP will emerge from strategies addressing local and systemic inflammation (3).

While specific targeting and modulation of the inflammation during AP will require detailed understanding of its pathogenesis (6), it may be possible to evaluate and repurpose various antiinflammatory strategies for their therapeutic potential in AP. In this regard, pirfenidone is an antiinflammatory and antifibrotic drug approved for the treatment of idiopathic pulmonary fibrosis (IPF) (7). While its exact mechanism of action is still unknown, pirfenidone has been shown to affect production of TGF-β (8) and its effect on downstream targets. It has also been shown to affect production of inflammatory mediators like TNF-α and ILs (9). Given its antiinflammatory effects in models of inflammation, we evaluated its therapeutic potential in models of AP and also evaluated the mechanism by which it modulates AP. Our studies demonstrate that pirfenidone is effective in abrogating the progression of moderate to severe AP in preclinical models. We delineate its mechanism of action and demonstrate that it affects cytokine production and halts the inflammatory cascade in acinar and immune cells. We also demonstrate that pirfenidone modulates the crosstalk between injured acinar cells and recruited immune cells and, thus, affects the progression of AP. Our study also suggests that most of the effects of pirfenidone are mediated through increased secretion of antiinflammatory cytokine IL-10 from macrophages.

## Results

### Prophylactic pirfenidone treatment ameliorates local pancreatic injury in a caerulein mouse model of AP.

To evaluate the efficacy of pirfenidone in AP, we first assessed its effect on the severity of caerulein AP when administered in a prophylactic fashion. The schematic of this study is shown in Supplemental Figure 1A (supplemental material available online with this article; https://doi.org/10.1172/jci.insight.141108DS1). As seen on H&E-stained sections, when administered prophylactically, pirfenidone reduced the severity of caerulein AP (Supplemental Figure 1B). Quantification of pancreatitis severity on H&E confirmed these findings (Supplemental Figure 1B). The reduction in the severity of AP by pirfenidone treatment was also reflected in reduced levels of serum amylase (Supplemental Figure 1C). Pirfenidone treatment reduced the inflammatory infiltrates, as measured by coronin staining, which stains all leukocytes, and by pancreatic myeloperoxidase (MPO) (Supplemental Figure 1D). We also measured the effect of pirfenidone on lung injury, as a measure of systemic inflammation. As seen in Supplemental Figure 1E, pirfenidone treatment reduced the lung injury, as seen on H&E-stained sections. Pirfenidone treatment reduced both pulmonary infiltrates, as well as alveolar thickness. Pirfenidone treatment reduced pulmonary inflammation, as measured by coronin staining and by lung MPO measurements (Supplemental Figure 1F). Pirfenidone alone, when administered to control mice (without AP), does not affect serum amylase, pancreas MPO, pancreas wet to dry weight ratio, lung MPO, or lung wet/dry weight ratio (Supplemental Figure 1, G–K).

### Therapeutic pirfenidone treatment ameliorates local pancreatic injury in a caerulein mouse model of severe AP.

**Figure 1:**
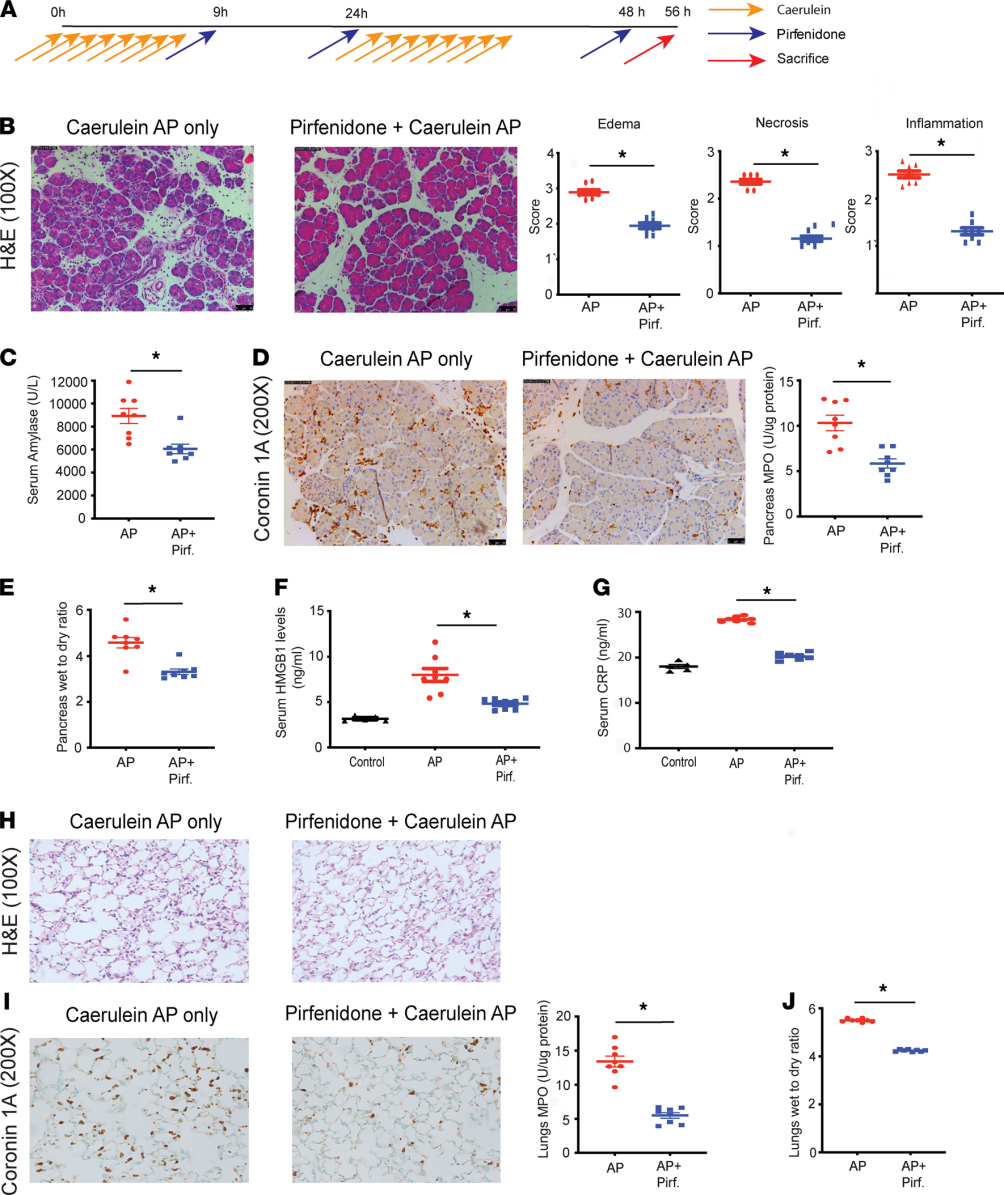
Therapeutic pirfenidone administration reduces local pancreatic injury and lung injury in caerulein 2-day model of acute pancreatitis. (**A**) Schematic of therapeutic administration of pirfenidone in a caerulein 2-day model of acute pancreatitis. (**B**) Representative histology (H&E, 100×) and histological analysis of pancreatic histology from AP-only group and pirfenidone-treated group. Therapeutic pirfenidone treatment leads to a decrease in pancreatic edema, necrosis, and inflammatory infiltrates. Histologic quantification of edema, necrosis, and inflammation is also shown. (**C**) Serum amylase levels were significantly decreased in the pirfenidone treatment group. (**D**) IHC for coronin 1A (200×), which stains leukocytes, shows a decrease in immune cell infiltration with pirfenidone treatment. Pancreatic MPO, which is a marker of neutrophilic infiltration, also shows a significant decrease with treatment. (**E**) Pancreas wet/dry weight ratio, a measure of pancreatic edema, shows a significant decrease in the pirfenidone-treatment group. (**F** and **G**) Serum HMGB1 and serum CRP, biomarkers that correlate with severity of AP, were significantly reduced with therapeutic pirfenidone treatment. (**H**) Lung H&E (100×) shows a reduction in injury with treatment. (**I**) IHC of lung sections for coronin 1A (200×) shows a decrease in immune infiltration with pirfenidone treatment. Lung MPO (myeloperoxidase) also shows a significant decrease with treatment. (**J**) Lung wet/dry weight ratio (a measure of pulmonary edema) shows a significant decrease in the treatment group. Pirf., pirfenidone. *n* = 8 each in AP-only and AP + pirf group. *n* = 5 each in control groups in **F** and **G**. Data represent mean ± SEM. *P < 0.05 by Mann-Whitney *U* test for **B**–**E**, **I**, and **J** and Kruskal-Wallis test (Dunn’s multiple-comparison test) for **F** and **G**.

Since prophylactic administration of potentially novel agents for the therapy of AP does not recapitulate the clinical scenario where patients present for medical care when the AP has already advanced to the systemic inflammation phase, we evaluated the efficacy of pirfenidone in the treatment of AP using therapeutic models. The schematic of this experiment, where the ability of therapeutically administered pirfenidone in reducing severity of 2-day caerulein AP was evaluated, is shown in Figure 1A. As seen in Figure 1B, therapeutically administered pirfenidone reduced inflammation, necrosis, and edema, as observed on H&E-stained sections in a 2-day caerulein AP model. Quantification of these parameters is also shown. That therapeutically administered pirfenidone reduced the severity of AP is also reflected in reduced serum amylase in the treatment arm (Figure 1C). Pirfenidone treatment also reduced local pancreatic inflammation, as measured by coronin IHC and pancreatic MPO (Figure 1D). Reduction of local pancreatic inflammation was further evident by reduced pancreatic edema, as measured by pancreas wet/dry weight ratio (Figure 1E). The effect of therapeutic administration of pirfenidone on systemic inflammation was also evaluated in this 2-day caerulein AP model. As seen in Figure 1, F and G, pirfenidone treatment reduced serum HMGB1 and CRP, 2 markers of inflammation that have been shown to correlate with adverse outcomes in clinical studies (10, 11). Pirfenidone, when administered therapeutically, is also able to reduce systemic injury and inflammation. As seen in Figure 1H, therapeutic pirfenidone treatment reduces lung injury in a 2-day caerulein model, as seen by reduced inflammatory infiltrates and alveolar thickness on H&E. Reduced lung inflammation by therapeutic pirfenidone treatment was also evident by reduced leukocyte infiltration on coronin staining and reduced lung MPO (Figure 1I), as well as reduced lung edema (as evident by reduced lung wet/dry weight ratio; Figure 1J).

### Pirfenidone attenuates local injury, inflammation, and associated lung injury in an L-arginine mouse model of AP.

To rule out any model-specific effect, we confirmed the ability of pirfenidone, when administered therapeutically, to attenuate local and systemic injury during AP in L-arginine model (Supplemental Figure 2A). As seen in Supplemental Figure 2B, L-arginine–induced pancreatitis is characterized by severe acinar cell necrosis, leukocyte infiltration, and edema. Pirfenidone, administered 36 hours after initiation of L-arginine AP, significantly improved all parameters of pancreatic injury (Supplemental Figure 2B). Quantification of the pancreatic injury supported this conclusion (Supplemental Figure 2B). Leukocyte infiltration during L-arginine–induced pancreatitis, as measured by IHC for coronin, also showed significant reduction with pirfenidone treatment (Supplemental Figure 2C). As seen in Supplemental Figure 2C, pirfenidone resulted in significant reduction in neutrophil recruitment to pancreas, as evaluated by measuring pancreatic MPO, following induction of L-arginine pancreatitis. Pirfenidone also led to a significant reduction in serum amylase compared with animals with L-arginine pancreatitis alone, indicating reduction of pancreatic injury (Supplemental Figure 2D). Pirfenidone reduced serum CRP, as well, suggesting a reduction of systemic inflammation (Supplemental Figure 2E).

Histologic analysis of lungs from mice with L-arginine–induced pancreatitis showed increased alveolar septal thickness and inflammatory infiltration (Supplemental Figure 2F) and increased leukocytic infiltration in the lung tissue (coronin IHC; Supplemental Figure 2G). Therapeutic pirfenidone resulted in a significant reduction in lung tissue injury in L-arginine AP, as shown by reduction in alveolar septal thickness and leukocyte infiltration (Supplemental Figure 2, F and G). Furthermore, Pirfenidone treatment also resulted in a significant reduction in neutrophil recruitment to the lungs in the L-arginine model, as represented by a significant reduction in lung MPO (Supplemental Figure 2G).

### Effect of pirfenidone on early events of AP.

**Figure 2:**
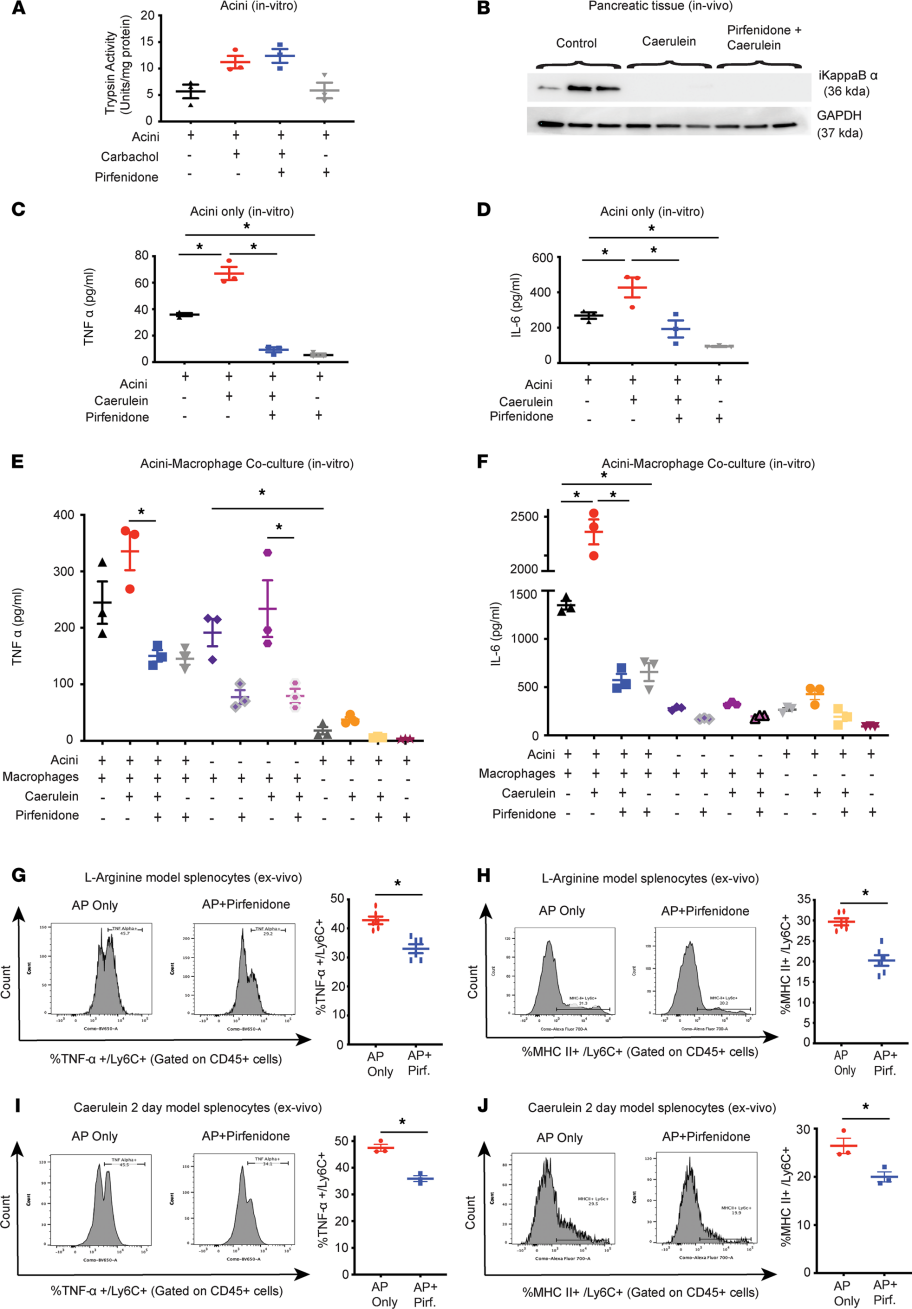
Evaluation of the effect of pirfenidone on early events in acute pancreatitis. Pancreatic acini were isolated from healthy C57BL/6J mice and treated with supramaximal carbachol, with or without pirfenidone pretreatment (0.5 mg/mL for 30 minutes before supramaximal stimulation) in vitro. (**A**) Treatment of pancreatic acini with supramaximal carbachol (1 mM) leads to trypsin activation. Pirfenidone pretreatment is unable to inhibit supramaximal carbachol–induced trypsin activation (*n* = 3). (**B**) Treatment with caerulein in vivo (50 μg/kg, i.p) results in IκBα degradation at 1 hour, as shown by Western blotting in pancreatic tissue. Pretreatment with pirfenidone gavage (400 mg/kg, 30 minutes prior to caerulein injection) does not affect IκBα degradation (*n* = 3). (**C** and **D**) Pretreatment with pirfenidone resulted in decreased secretion of TNF-α (**C**) and IL-6 (**D**) from acini treated with supramaximal caerulein (100 nM) for 4 hours (*n* = 3). (**E** and **F**) Pirfenidone disrupts acinar cell–macrophage crosstalk. Pretreatment with pirfenidone resulted in attenuation of the acinar cell mediated activation of macrophages as shown by decreased secretion of TNF-α (**E**) and IL-6 (**F**) (*n* = 3). (**G**–**J**) Splenocytes isolated from C57BL/6J mice with L-arginine AP (**G** and **H**) (*n* = 6) or caerulein 2-day model AP (**I** and **J**) (*n* = 3) at the peak of injury were cultured in duplicate, with or without pirfenidone (0.5 mg/mL) for 12 hours, and cells were analyzed using flow cytometry. Pirfenidone treatment led to a significant decrease in TNF-α^+^Ly6C^+^ cells (**G** and **I**) and MHCII^+^ Ly6C^+^ cells (**H** and **J**) in the L-arginine model, as well as caerulein 2-day model. Pirf., pirfenidone. Data represent mean ± SEM. *P <.05 by ordinary 1-way ANOVA for (**C** and **D**); 2-way ANOVA for (**E** and **F**), and Mann-Whitney *U* test for (**G**–**J**).

To elucidate the mechanism by which pirfenidone affects severity of AP, we systematically evaluated its effect on early events in AP. Since trypsin activation and NF-κB activation are key early events of AP, we studied the effect of pirfenidone on these events. Briefly, acini were treated in vitro with pirfenidone (0.5 mg/mL) for 30 minutes before being stimulated with supramaximal carbachol (1 mM). As seen in Figure 2A, as expected, carbachol led to trypsin activation. However, pirfenidone was unable to inhibit carbachol-induced trypsin activation. We next looked at the effect of pirfenidone on NF-κB activation during AP (in vivo). NF-κB activation is a multistep process and involves IκBα degradation and release of p65 and p50 subunits, which then translocate to the nucleus and bind to NF-κB response elements in various genes regulated by NF-κB. Thus, we evaluated whether pirfenidone influences IκBα degradation by immunoblotting. As shown in Figure 2B, in vivo stimulation with caerulein leads to NF-κB activation, as evident by IκBα degradation at 1 hour, and pirfenidone pretreatment 30 minutes before giving caerulein is not useful in preventing this. This suggests that pirfenidone, most likely, is unable to prevent p65 translocation to the nucleus. However, it has been shown previously that pirfenidone inhibits the DNA binding of p65 to NF-κB response elements in hepatocytes in response to IL-1β (12). Hence, we evaluated the effect of pirfenidone (0.5 mg/mL) on the binding of p65 subunit of NF-κB to DNA in pancreatic acinar cells treated with supramaximal dose of caerulein (100nM) in vitro for 1 hour (Supplemental Figure 3I) or 3 hours (Supplemental Figure 3J). Nuclear extracts were analyzed by electrophoretic mobility shift assay (EMSA), which showed that pirfenidone reduced NF-κB DNA binding at the 3-hour but not at the 1-hour incubation period.

Since pirfenidone is able to decrease the inflammation in AP, we next evaluated the effect of pirfenidone on secretion of cytokines from acini and macrophages and acinar-macrophage crosstalk. As seen in Figure 2, C and D, pretreatment with pirfenidone decreases TNF-α and IL-6 secretion from the acini stimulated with supramaximal caerulein (100 nM). Next, we evaluated the effect of pirfenidone on acinar-macrophage crosstalk and secretion of cytokines. As seen in Figure 2, E and F, coculture of caerulein-stimulated acini and macrophages leads to markedly more TNF-α and IL-6 in the culture medium, when compared with either acini or macrophage alone, suggesting an ongoing crosstalk between pancreatic acini and macrophages. Pirfenidone pretreatment abrogated this crosstalk and decreased secretion of TNF-α and IL-6 in the coculture medium. While this experiment suggests that pirfenidone treatment affects acinar cell–macrophage crosstalk, it does not illustrate which cell is responsible for the increased secretion of these cytokines in a coculture system or whether it is a direct or indirect effect of pirfenidone. To evaluate these aspects of the crosstalk further, we have used following 2 additional approaches.

As seen in Supplemental Figure 3, A and B, in our first approach, we pretreated pancreatic acinar cells with caerulein, with or without pirfenidone, for 1 hour. After this, the acinar cells were washed 3 times with media and cocultured with macrophages for 3 hours, and the effects of these activated acinar cells, treated with or without pirfenidone, on macrophages were evaluated. This was different from our experiment shown in Figure 2, E and F, as the pirfenidone was not in the coculture system, and the acinar cells were only pretreated with it. As seen in Supplemental Figure 3, A and B, coculture with caerulein-pretreated acini leads to increased TNF- α and IL-6 secretion in the coculture. Coculture with caerulein and pirfenidone pretreated acini did not lead to this change, which suggests that there was a crosstalk between injured (caerulein pretreated) acini and the macrophages, and this was prevented by pirfenidone pretreatment. We understand that the secreted TNF-α and IL-6 in this coculture could be coming from macrophages and/or acini. We believe that the majority of this is from the macrophages, since when comparing acini and macrophages (Figure 2), it appears that acini alone are not major sources of these cytokines by themselves.

In our second approach, instead of coculturing the acini and the macrophages, we transferred the culture media supernatant from acini — which were treated with supramaximal caerulein, with or without pirfenidone for 3 hours — on to macrophages and looked for cytokine secretion after another 3 hours. As seen in Supplemental Figure 3, C and D, treatment of macrophages, with culture media of caerulein stimulated acinar cells, led to increased TNF-α and IL-6 secretion. Treatment of macrophages with medium from caerulein stimulated, but pirfenidone pre-treated acinar cells, prevented this increase in TNF-α and IL-6 secretion. This experiment shows that the macrophage activation is due to factors released by acinar cells and that pirfenidone abrogates acinar cell–macrophage crosstalk. Our results also suggest that pirfenidone reduces TNF-α and IL-6 levels at a translational level or at the level of cytokine release — and not at transcriptional levels (Supplemental Figure 3, E and F). Furthermore, pirfenidone does not affect the viability of macrophages at the dose used for the above-mentioned in vitro studies (0.5 mg/mL) (Supplemental Figure 3, G and H).

We next evaluated the effect of pirfenidone treatment on proinflammatory phenotype of immune cells in AP. Briefly, splenocytes were isolated from mice with L-arginine AP at 72 hours (Supplemental Figure 2A) or a caerulein 2-day AP model at 32 hours (Figure 1A) at the peak of injury, and they were cultured with or without pirfenidone for 12 hours, followed by flow cytometry analysis. As seen in Figure 2, G–J, pirfenidone treatment significantly inhibited TNF-α production and MHCII expression by the monocytes, suggesting decreased proinflammatory phenotype.

### Effect of pirfenidone on immune environment and kinetics of recovery from a well-established model of L-arginine–induced AP.

**Figure 3:**
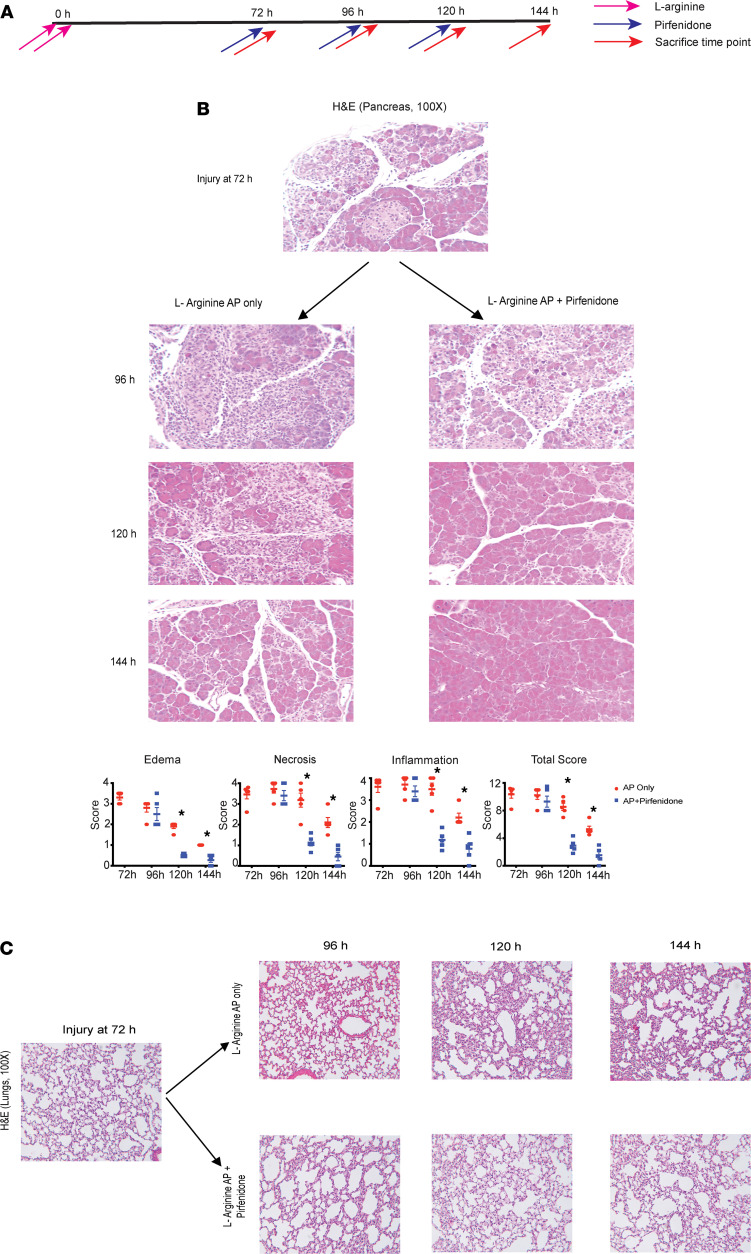
Therapeutic pirfenidone, when administered at the peak of injury in a well-established L-arginine model of acute pancreatitis, starts reducing local pancreatic and lung injury at 120 hours. (**A**) Schematic of pirfenidone administration in a therapeutic manner in well-established L-arginine model of acute pancreatitis. (**B**) Histological analysis of representative H&E sections (100×) of pancreata from AP-only group and pirfenidone-treated AP group. A decrease in pancreatic edema, necrosis, and inflammatory infiltrate can be seen. Histologic quantification of edema, inflammation, and necrosis is shown. (**C**) Lung H&E (100×) at 120 hours shows a reduction in injury with pirfenidone treatment. Pirf., pirfenidone. Data represent mean ± SEM and *n* = 5 in each group. *P < 0.05 by Mann-Whitney *U* test for each time point (as we are not comparing changes between time points).

**Figure 4:**
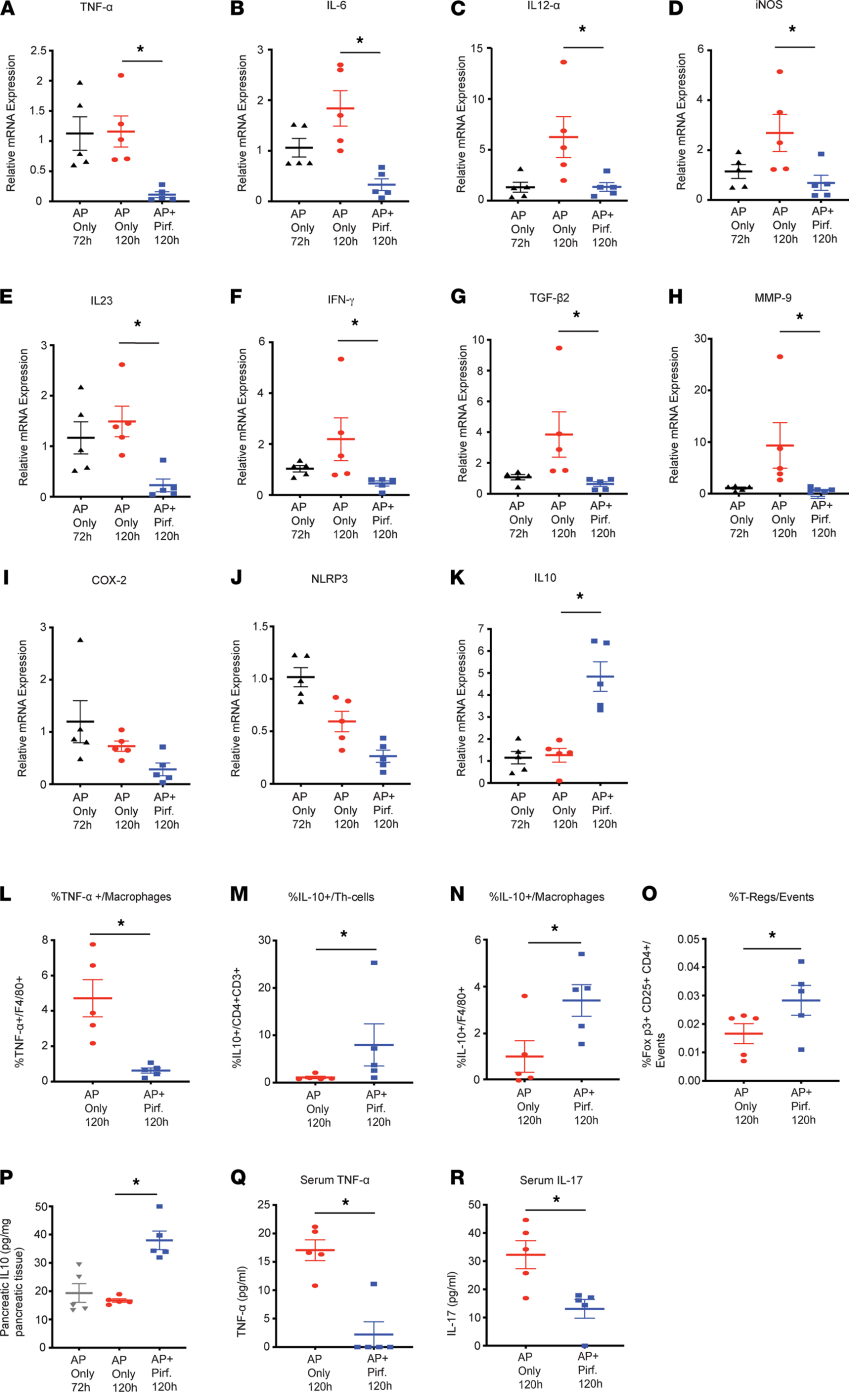
Therapeutic pirfenidone modulates immune and cytokine microenvironment at 120 hours, when histological changes start to appear in L-arginine model. (**A**–**K**) mRNA levels of proinflammatory cytokines TNF-α, IL-6, IL-12α, iNOS, IL-23, IFN-γ, TGF-β2, MMP-9, COX-2, and NLRP3 showed a significant decrease with pirfenidone treatment, while the antiinflammatory cytokine IL-10 was significantly increased with pirfenidone treatment. (**L**–**N**) Flow cytometry analysis of pancreatic immune cells at 120 hours demonstrates that pirfenidone treatment leads to a significant decrease in percentage of TNF-α secreting macrophages and a significant increase in percentage of IL-10 secreting Th cells and macrophages. (**O**) Pirfenidone treatment also led to statistically significant increase in Tregs. (**P**) Pirfenidone treatment significantly increased intrapancreatic IL-10 levels at 120 hours, as measured by ELISA. (**Q** and **R**) Multi-Analyte Flow Assay for cytokines TNF-α and IL-17 in serum demonstrates that pirfenidone is able to suppress the level of these cytokines. Data represent mean ± SEM and *n* = 5 in each group. *P < 0.05 by Kruskal-Wallis Test (Dunn’s multiple-comparison test) for **A**–**K** and **P** and Mann-Whitney *U* test for **L**–**O**, **Q**, and **R**.

To further dissect the mechanism by which pirfenidone attenuates severity of AP, we evaluated the early effects of therapeutically administered pirfenidone in L-arginine AP. The schematic of this experiment is shown in Figure 3A. Briefly, pirfenidone treatment was started at the peak of injury (72 hours after initiation) in L-arginine AP, after confirming AP induction with more than a 3-fold increase in serum amylase levels obtained by retro-orbital bleeding, simulating treatment of AP patients presenting to the clinics. The animals from the control (L-arginine AP only) and the pirfenidone-treated group were sacrificed every 24 hours, after starting treatment. Consistent with our other experiments where therapeutic pirfenidone treatment reduces severity of AP, in this therapeutic model as well, pirfenidone treatment reduced edema, necrosis, and inflammation during L-arginine AP (Figure 3B). Intriguingly, the salutary effect of pirfenidone on AP started within 48 hours of its administration. Similar to its effect on pancreatic injury and inflammation, therapeutic pirfenidone treatment improves lung injury and inflammation within 48 hours of starting treatment (120 hours of the experiment; Figure 3C). To further dissect out the mechanism of action of pirfenidone, we evaluated the effect of pirfenidone treatment on the message level of various cytokines and other inflammatory/antiinflammatory molecules in the pancreas at 120 hours, when we start to see improvement in histology. As seen in Figure 4, A–J, pirfenidone treatment decreased the message levels of proinflammatory molecules TNF-α, IL-6, IL-12α, iNOS, IL-23, IFN-γ, TGF-β2, MMP-9, COX-2, and NLRP3. Intriguingly, pirfenidone increased the message levels of IL-10, an antiinflammatory cytokine (Figure 4K). The cytokines that were not affected by pirfenidone treatment in a statistically significant fashion are shown in Supplemental Figure 4A.

We also performed detailed immune profiling on the pancreatic tissue for innate and adaptive immune cells. Pirfenidone treatment decreased levels of activated (TNF-α^+^) macrophages in the pancreas (Figure 4L). Supporting the message-level data, pirfenidone treatment increased the IL-10 secretion from Th cells, as well as macrophages (Figure 4, M and N). Pirfenidone also increased the levels of Tregs in the L-arginine AP pancreas (Figure 4O). The gating strategy for flow analysis at the 120-hour time point is shown in Supplemental Figure 4, B–E. IL-10 ELISA on pancreatic tissue at 120 hours confirmed a significant increase in IL-10 levels in the pancreas with pirfenidone treatment (Figure 4P). Multi-Analyte Flow Assay for cytokines TNF-α (Figure 4Q) and IL-17 (Figure 4R) in serum showed that they were reduced by pirfenidone treatment. The immune cells not affected by pirfenidone in a statistically significant fashion are shown in Supplemental Figure 5, A and B.

The beneficial effects of pirfenidone are established at 144-hour time point, when histological analysis of representative H&E sections of pancreas from the AP-only group and the pirfenidone-treated AP group show a decrease in pancreatic edema, necrosis, and inflammatory infiltrate (Supplemental Figure 6A). Lung H&E also shows a reduction in injury with pirfenidone treatment (Supplemental Figure 6B). Histologic quantification of edema, necrosis, and inflammation is shown, which confirms the salutary effects of pirfenidone (Supplemental Figure 6, C–F). The message levels of proinflammatory markers TNF-α, IL-6, IFN-γ, and IL-23 show a significant decrease with pirfenidone treatment, while IL-12α and NLRP3 show a decreasing trend (Supplemental Figure 6, G–L). Serum HMGB1 levels, a serum marker of necrosis, show a significant reduction at 144 hours with pirfenidone treatment (Supplemental Figure 6M).

**Figure 5:**
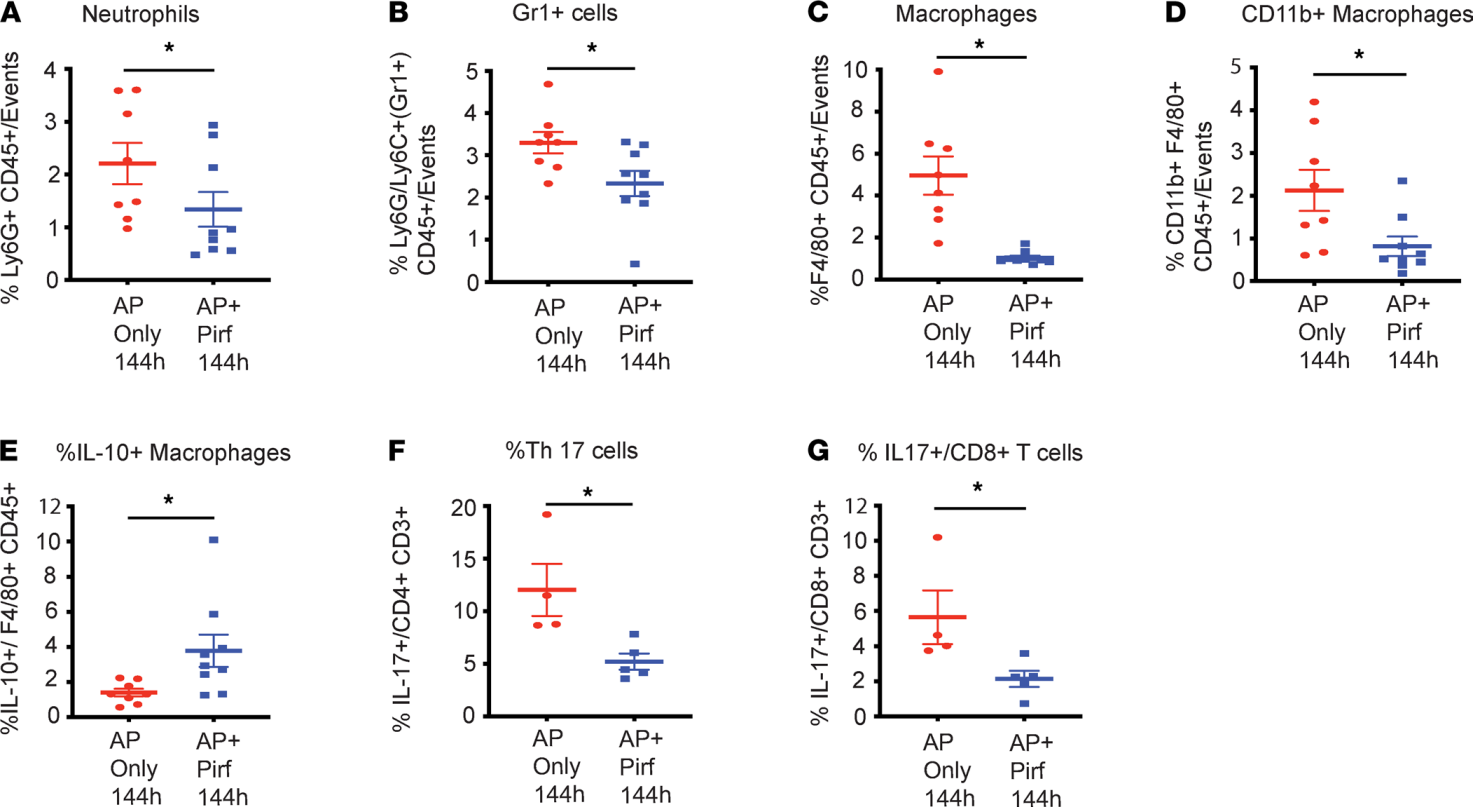
Therapeutic administration of pirfenidone modulates immune infiltration as well as immune cell phenotype in the well-established L-arginine model of acute pancreatitis at 144 hours. (**A**–**G**) Flow cytometry analysis of pancreatic immune cells at the 144-hour time point shows a reduction in infiltration of neutrophils (**A**), Gr1^+^ cells (**B**), and macrophages (**C**); pirfenidone also modulated the phenotype of immune cells with decreased intrapancreatic levels of activated macrophages (**D**) but increased levels of IL-10 secreting macrophages (**E**), and pirfenidone treatment led to decreased intrapancreatic abundance of IL-17 secreting Th (**F**) and cytotoxic T cells (**G**). Pirf., pirfenidone. *n* = 8–9 each in macrophage/neutrophil panel (*n* = 8 in AP-only group; *n* = 9 in AP + pirf group). *n* = 4–5 each in T cell panel (*n* = 4 each in AP-only group; *n* = 5 each in AP + pirf group). Data represent mean ± SEM. *P < 0.05 by Mann-Whitney *U* test.

To further elucidate the mechanism of pirfenidone’s effect, we evaluated its effect on pancreatic immune environment in L-arginine AP at the 144-hour time point (72 hours after initiation of pirfenidone). As seen in Figure 5, pirfenidone — in accordance with its salutary effects in AP — decreased the levels of neutrophils (Figure 5A), Gr1^+^ cells (Figure 5B), macrophages (Figure 5C), activated macrophages (Figure 5D), and IL-17–secreting Th cells (Th17; Figure 5F) and CD8 cells (Tc17; Figure 5G). IL-10–secreting macrophages were increased at this time point, also (Figure 5E). The gating strategy is shown in Supplemental Figure 7. The immune cells not affected by pirfenidone in a statistically significant fashion at 144 hours are shown in Supplemental Figure 5A.

### Therapeutic pirfenidone induced increase in IL-10 precedes changes in histology and the decrease in the proinflammatory and M1 markers.

**Figure 6:**
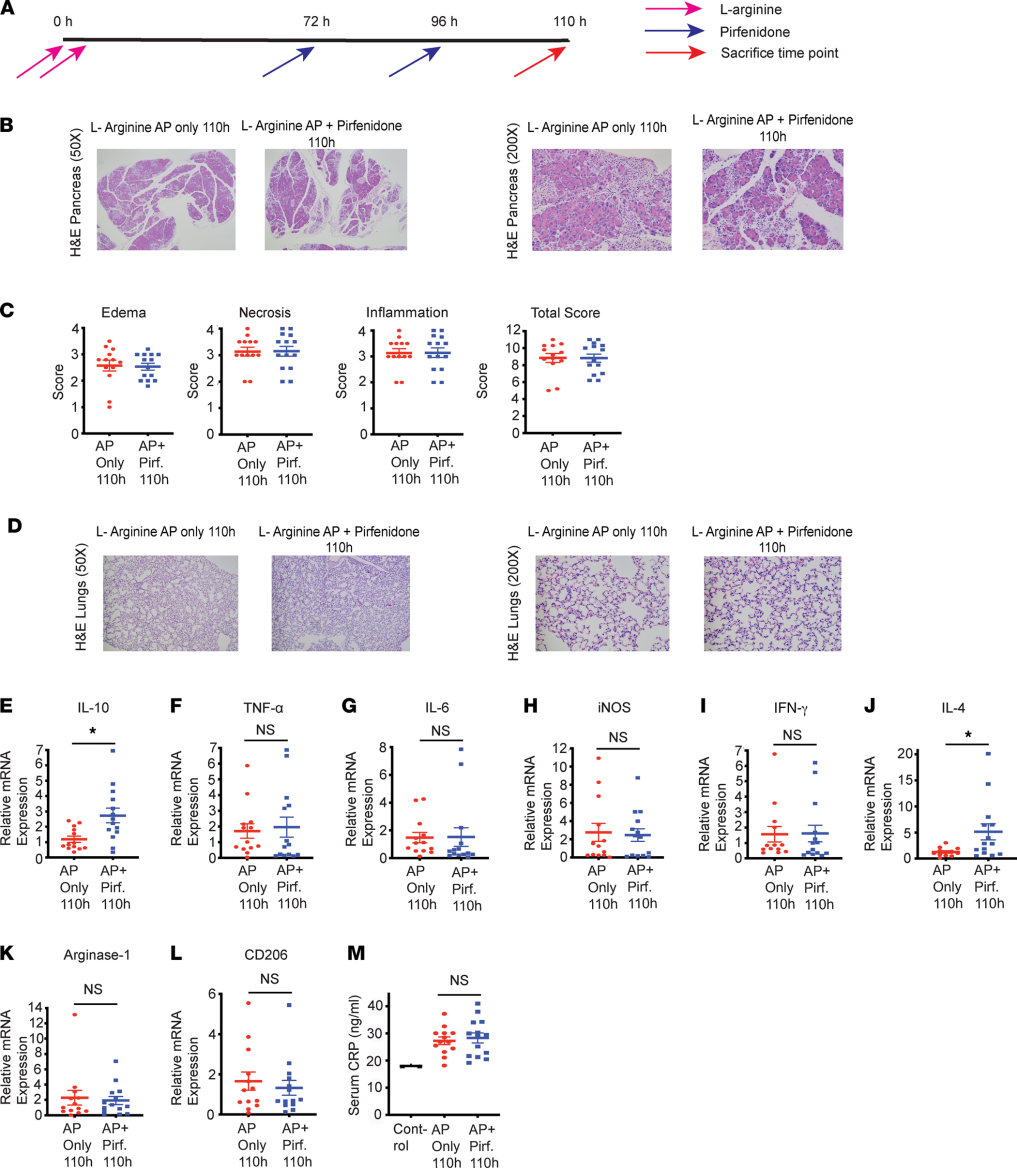
Therapeutic administration of pirfenidone increases IL-10 levels before the histology changes in L-arginine model. (**A**) Schematic of pirfenidone administration in a therapeutic manner in well-established L-arginine model of acute pancreatitis. (**B**) Histology of representative H&E sections (50× and 200×) of pancreas from AP-only group and pirfenidone-treated AP group at 110 hours after L-arginine injections is shown. (**C**) Histologic quantification of edema, necrosis, and inflammation is shown. Pirfenidone treatment does not change pancreatic edema, necrosis, and inflammatory infiltrate at 110 hours. (**D**) Lung H&E staining (50× and 200×) shows no reduction in injury with pirfenidone treatment at 110-hour time point. (**E**–**L**) mRNA levels of various cytokines is shown. mRNA levels of antiinflammatory cytokine IL-10 (**E**) was significantly increased with pirfenidone treatment at 110 h time point. (**F**–**L**) mRNA levels of TNF-α, IL-6, i NOS, IFN-γ, IL-4, Arginase-1, and CD206 did not change at 110 hours. (**M**) Serum CRP levels do not change with pirfenidone treatment at 110 hours. Pirf., pirfenidone. Data represent mean ± SEM (*n* = 13 in AP-only group; *n* = 14 in pirfenidone-treatment group). *P < 0.05 by Mann-Whitney *U* test for **C** and **E**–**L** and Kruskal-Wallis test (Dunn’s multiple-comparison test) for **M**.

**Figure 7:**
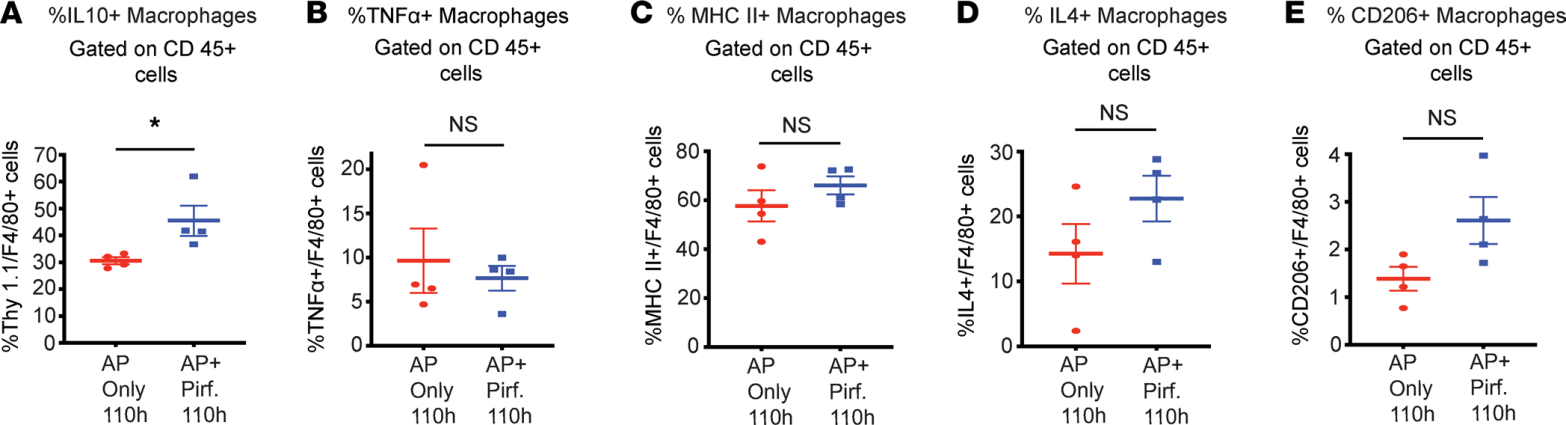
Therapeutic administration of pirfenidone increases IL-10–secreting macrophages in IL-10 reporter mice preceding changes in histology or reduction in TNF-α^+^ macrophages at 110 hours in the L-arginine model. (**A**) Flow cytometry analysis of pancreatic immune cells at 110-hour time point shows a significant increase in the levels of IL-10–secreting macrophages in IL-10 reporter mice (C57BL/6 background). (**B** and **C**) TNF-α^+^ and MHCII^+^ M1 macrophages did not show any change with treatment at this time point. (**D** and **E**) IL-4^+^ and CD206^+^ M2 macrophages showed an increasing trend with treatment, but it was not statistically significant. Pirf., pirfenidone. Data represent mean ± SEM and *n* = 4 in each group. *P < 0.05 by Mann-Whitney *U* test.

We have observed that pirfenidone treatment leads to increased level of antiinflammatory cytokine IL-10 at 120 hours or 48 hours after starting the pirfenidone treatment in a well-established model of L-arginine–induced AP. As IL-10 is also involved in wound healing in general (13), it is possible that the increase in IL-10 observed with pirfenidone treatment is a result of improved AP severity rather than a direct effect of pirfenidone treatment. Thus, to differentiate between these possibilities and to better understand the role of increased IL-10 in improvement of AP mediated by pirfenidone, we evaluated the effect of pirfenidone on pancreatic IL-10 levels, cytokine profile, and immune environment at a time point when the pancreatic histology has not started showing any change in response to pirfenidone treatment. For this, we sacrificed the experimental animals at 110 hours after the first L-arginine injection, several hours before changes in histology (schematic shown in Figure 6A). As seen in Figure 6, pancreatic (Figure 6, B and C) and lung injury (Figure 6D) do not show any improvement with pirfenidone treatment at 110 hours. Interestingly, the message levels of IL-10 was significantly increased with pirfenidone treatment at 110 hours, although TNF-α, IL-6, iNOS, IFN-γ, Arginase-1, and CD206 did not change (Figure 6, E–L). As a serum marker of inflammation and tissue injury, CRP also did not show any change with treatment at this time point (Figure 6M). Immune characterization at this time point was done in IL-10 reporter mice (C57BL/6J background) (14). As seen in Figure 7, pirfenidone increased IL-10–secreting macrophages significantly (Figure 7A) without a change in M1 markers, TNF-α (Figure 7B) and MHCII (Figure 7C), as early as 36 hours after starting treatment with pirfenidone. Other M2 markers, IL-4 (Figure 7D) and CD206 (Figure 7E), showed an increase, but it was not statistically significant. The gating strategy is shown in Supplemental Figure 8. Pirfenidone does not affect total macrophage infiltration, neutrophil infiltration, or T cells at this time point (Supplemental Figure 9). These data suggest that an increase in IL-10 levels is the earliest change induced by pirfenidone treatment in experimental animals with AP.

### Pirfenidone improves AP in an IL-10–dependent fashion in a well-established model of L-arginine–induced AP.

**Figure 8:**
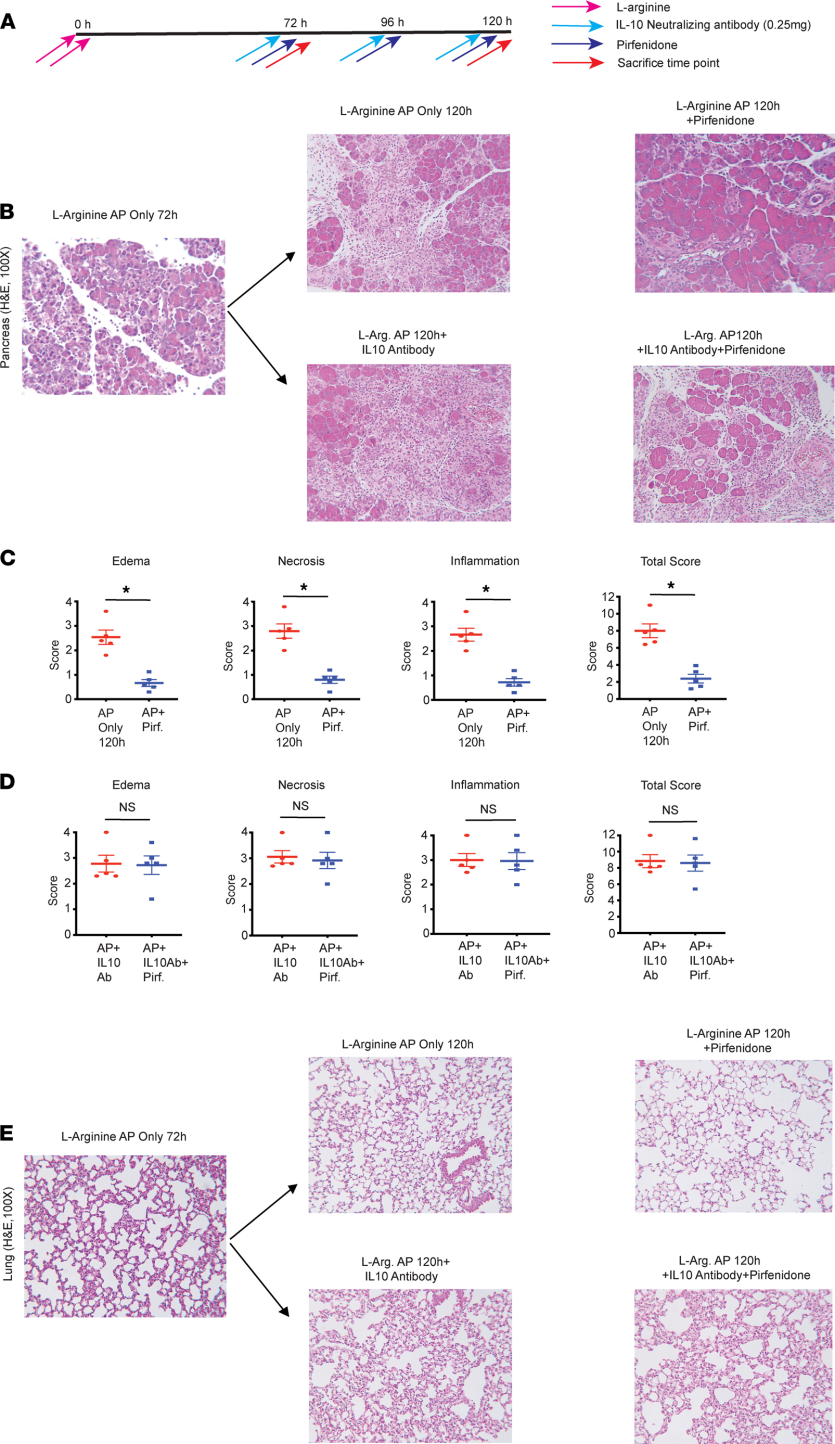
Therapeutic pirfenidone improves pancreatic and lung injury in the L-arginine model of acute pancreatitis in an IL-10–dependent fashion. (**A**) Schematic of pirfenidone administration in a therapeutic manner in L-arginine model of acute pancreatitis with well-established disease, with or without IL-10 neutralizing antibody, is shown. (**B**) Histology of representative H&E sections (100×) of pancreata from AP-only group, as well as pirfenidone-treated AP group, with or without IL-10 neutralization, is shown. (**C**) Pirfenidone starts improving L-arginine–induced acute pancreatitis at 120 hours. (**D**) Pirfenidone is not able to improve L-arginine induced AP when IL-10 is neutralized with antibodies, as evident by unchanged edema, necrosis, and inflammation scores. (**E**) Lung H&E (100×) shows a reduction in injury with pirfenidone treatment but not in the presence of IL-10 neutralization. Pirf., pirfenidone; IL-10 Antibody, IL-10 neutralizing antibody. Data represent mean ± SEM and *n* = 5 in each group. *P < 0.05 by Mann-Whitney *U* test.

**Figure 9:**
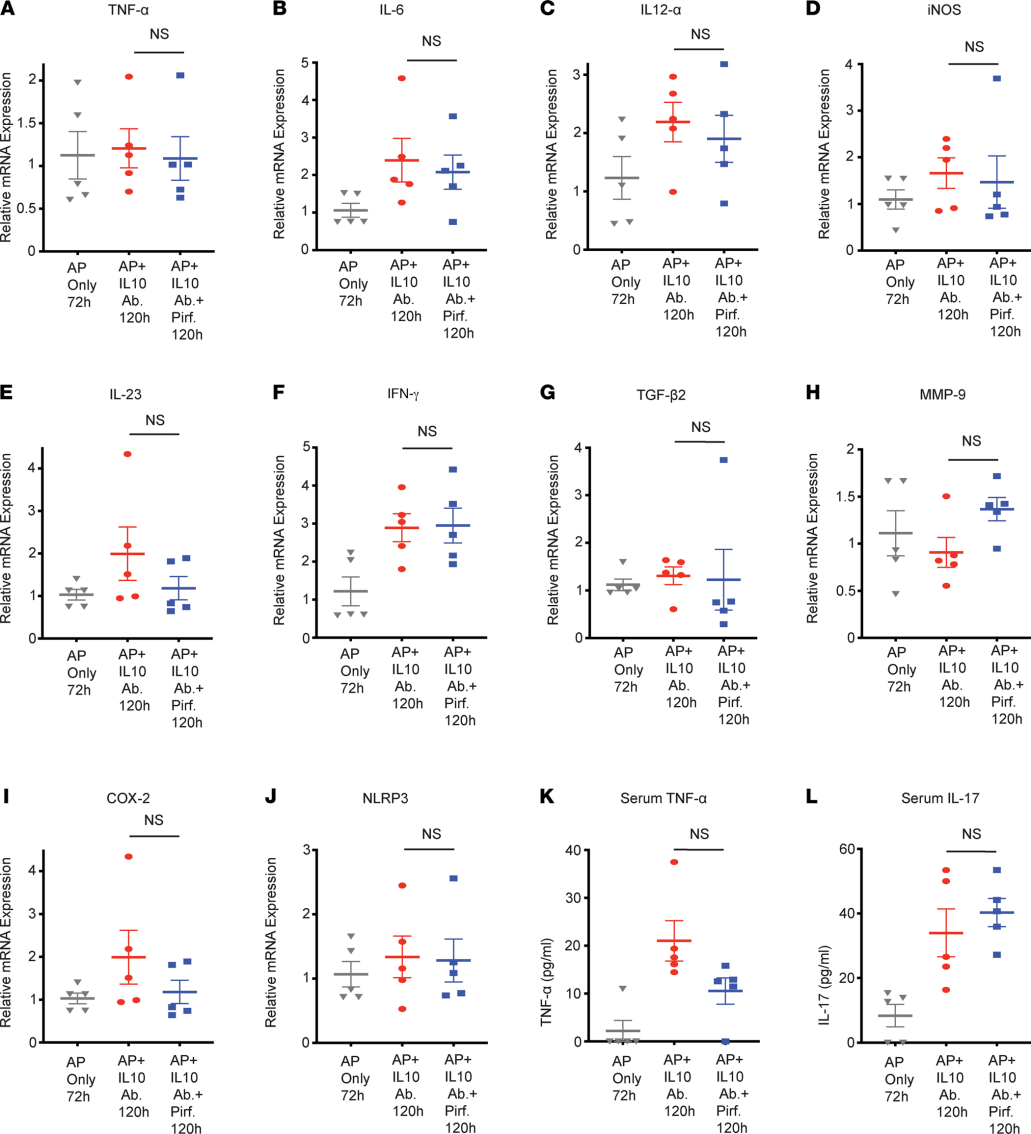
Therapeutic pirfenidone does not reduce proinflammatory cytokines in the absence of IL-10 in the L-arginine model. (**A**–**J**) Quantification of the mRNA levels of TNF-α, IL-6, IL-12α, iNOS, IL-23, IFN-γ, TGF-β2, MMP-9, COX-2, and NLRP3 demonstrates that pirfenidone is unable to reduce their levels in the presence of IL-10 neutralization. (**K** and **L**) Multi-Analyte Flow Assay for cytokines TNF-α and IL-17 in serum is in agreement with the notion that pirfenidone is not able to reduce their levels in the presence of IL-10 neutralization. Pirf., pirfenidone; IL-10 Ab, IL-10 neutralizing antibody. Data represent mean ± SEM and *n* =5 in each group. *P < 0.05 by Kruskal-Wallis test (Dunn’s multiple-comparison test).

As discussed above, pirfenidone treatment increased pancreatic infiltration of IL-10–secreting macrophages and increased intrapancreatic IL-10 mRNA at a time point at which the histology does not change. Given that IL-10 is an antiinflammatory cytokine, we hypothesized that pirfenidone exerts its antiinflammatory action by increasing IL-10 secretion. To test this hypothesis, we evaluated the ability of pirfenidone in improving L-arginine AP in the absence of IL-10. For this, mice with L-arginine AP were randomized into the following groups (a) L-arginine AP only; (b) L-arginine AP with antibody-mediated IL-10 depletion; (c) L-arginine AP with pirfenidone treatment; and (d) L-arginine AP with IL-10 depletion and pirfenidone treatment (schematic is shown in Figure 8A). As seen in Figure 8, B and C, and similar to the experiment in Figure 3, pirfenidone treatment leads to improvement after 48 hours of administration. Intriguingly, as seen in Figure 8, B and D, pirfenidone is unable to improve L-arginine AP in the absence of IL-10, suggesting that some, if not all, of its effects are mediated by increased IL-10 secretion. The quantification of various parameters of injury is also shown. Also, and in accordance with the hypothesis that the antiinflammatory effects of pirfenidone are mediated via IL-10, pirfenidone was unable to improve lung injury and inflammation in the absence of IL-10 (Figure 8E). Similarly, therapeutic pirfenidone treatment was unable to decrease the message levels of inflammatory cytokines and molecules in the pancreas in the absence of IL-10 (Figure 9, A–J). Multi-Analyte Flow Assay for cytokines TNF-α (Figure 9K) and IL-17 (Figure 9L) in serum demonstrated an inability of pirfenidone to decrease these cytokines in the absence of IL-10. Please note that pirfenidone suppresses TNF-α at the translational level (15).

### Pirfenidone reprograms macrophages (in vitro) to an antiinflammatory M2 phenotype in proinflammatory M1 polarizing conditions.

**Figure 10:**
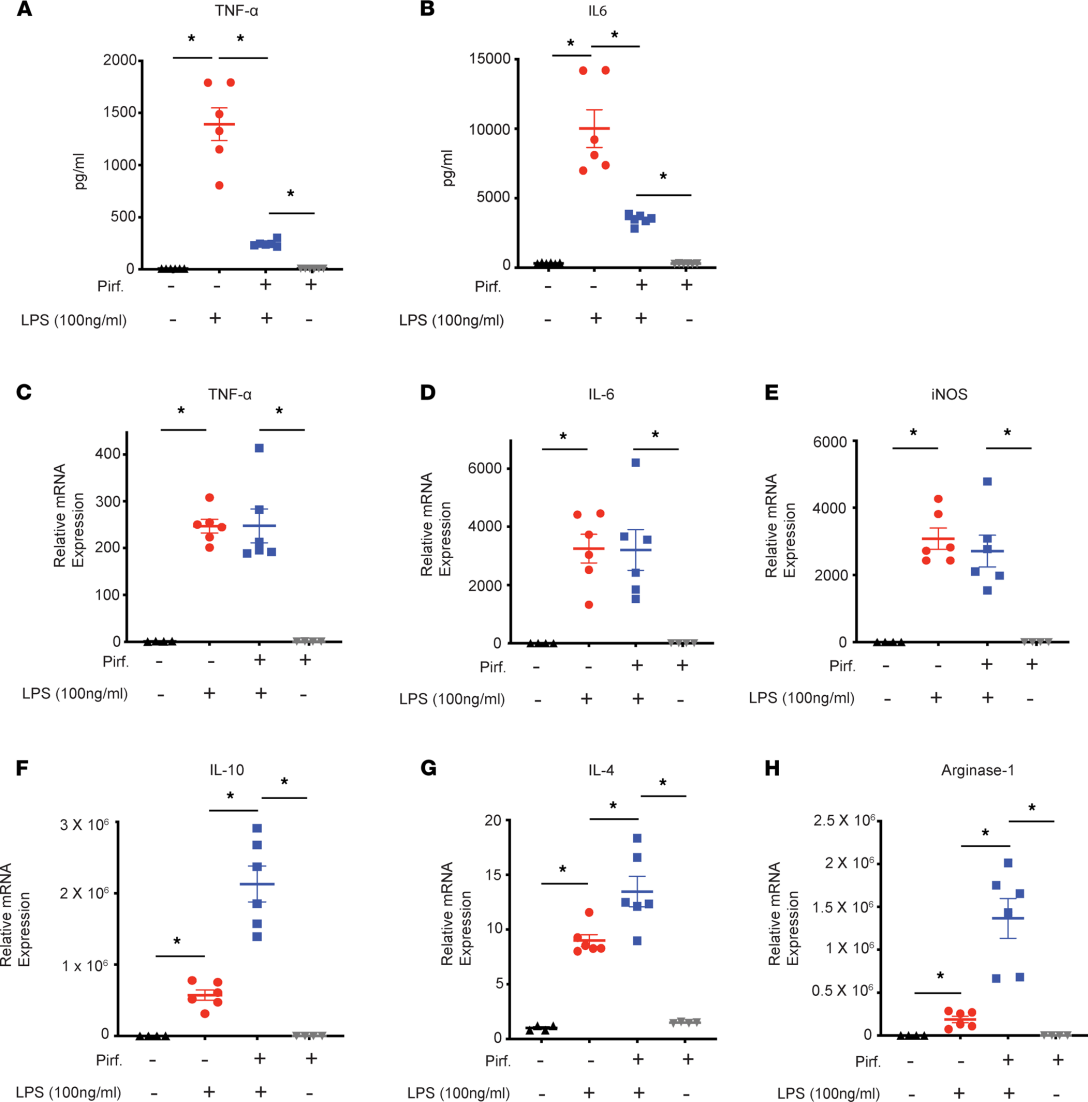
Pirfenidone treatment of macrophages in M1-polarizing conditions increases IL-10 and other M2 markers. BMDMs were isolated from healthy C57BL/6J mice and treated with LPS 100 ng/mL (M1 polarizing conditions), with or without pirfenidone treatment (0.5 mg/mL) and incubated for 24 hours. (**A**–**E**) Pirfenidone significantly reduces M1 markers TNF-α and IL-6 secretion from macrophages at protein levels (**A** and **B**) but not at mRNA levels (**C** and **D**); iNOS (M1) (**E**) is also not affected at mRNA levels with pirfenidone. (**F**–**H**) Pirfenidone significantly increases M2 markers, viz IL-10 (**F**), IL-4 (**G**), and Arginase-1 (**H**) in M1 polarizing conditions. Pirf., pirfenidone. For **A** and **B**, *n* =6 in each group. For **C**–**H**, *n* = 6 in each group treated with LPS, and *n* = 4 in macrophage-only and macrophage + pirf. groups. Data represent mean ± SEM *P < 0.05 by ordinary 1-way ANOVA for **A** and **B** and Kruskal-Wallis test (Dunn’s multiple-comparison test) for **C–H**.

To decipher the effect of pirfenidone on macrophage polarity in vitro, we treated macrophages with LPS (100 ng/mL) with or without pirfenidone (0.5 mg/mL) for 24 hours. M1 markers — e.g., TNF-α and IL-6 — reduced significantly at protein levels (Figure 10, A and B) in the supernatant but not at mRNA levels (Figure 10, C and D). iNOS also did not change at message levels (Figure 10E). Intriguingly, IL-10 increased significantly at mRNA levels (Figure 10F). Other M2 markers — e.g., IL-4 and Arginase 1 — also increased significantly with pirfenidone treatment (Figure 10, G and H) denoting a shift to an antiinflammatory M2 phenotype in proinflammatory M1 polarizing conditions.

### Therapeutic pirfenidone does not ameliorate pancreatitis in IL-10–KO mice or when macrophages are depleted using liposomal clodronate in a well-established model of L-arginine–induced AP.

**Figure 11:**
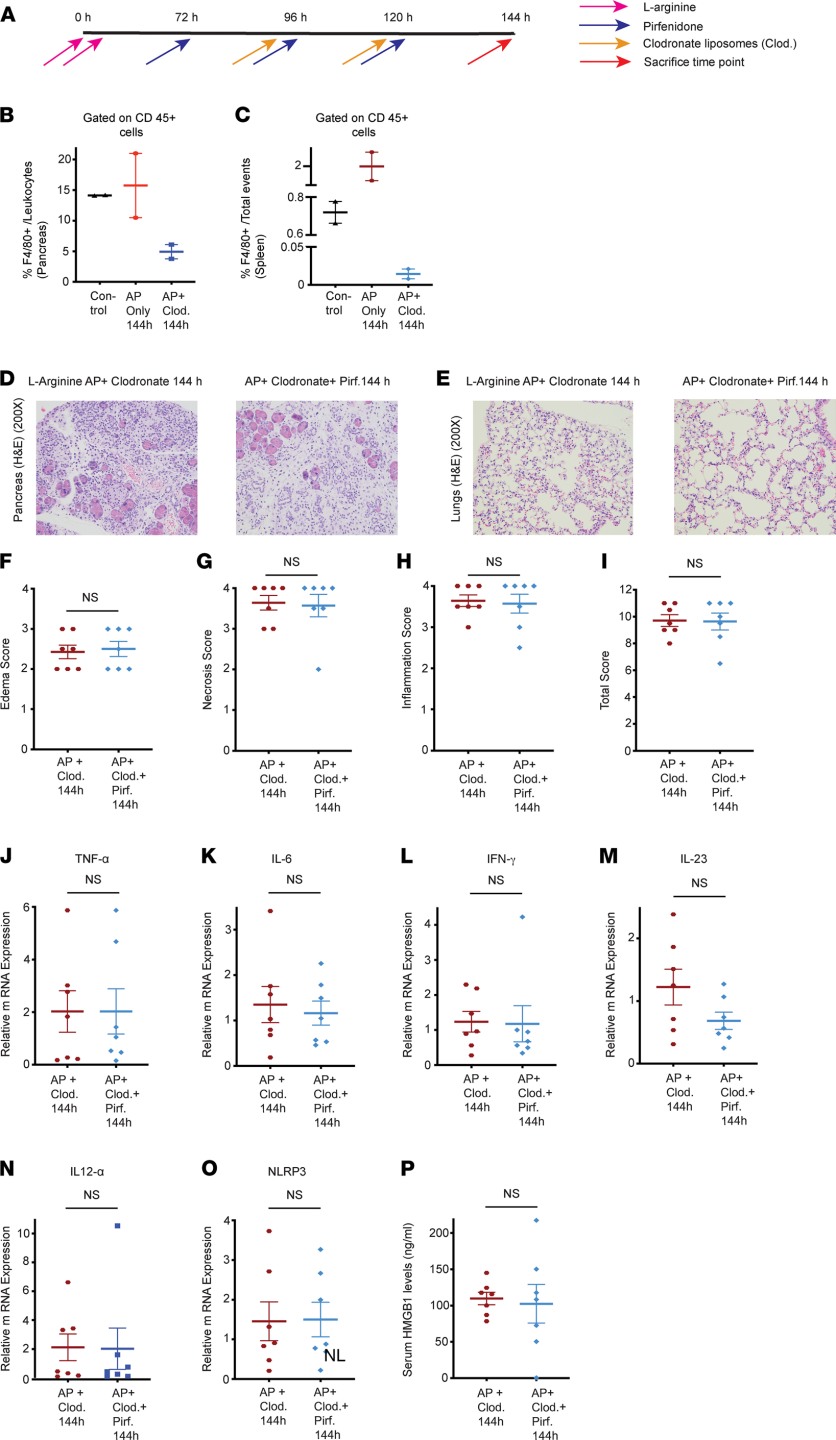
Depletion of macrophages with clodronate liposomes abrogates the beneficial effects of therapeutic pirfenidone in L-arginine AP. (**A**) Schematic of pirfenidone administration in a therapeutic manner in well-established L-arginine model of acute pancreatitis with clodronate liposomes (i.p., 200 μL) administration at 96 and 120 hours. (**B** and **C**) Confirmation of macrophage depletion using clodronate in pancreas (**B**) and spleen (**C**). (**D**) Histological analysis of representative H&E sections (200×) of pancreas from AP-only group and pirfenidone-treated AP group. There is no decrease in pancreatic edema, necrosis, and inflammatory infiltrate with pirfenidone treatment with depletion of macrophages. (**E**) Lung H&E (200×) does not show a reduction in injury with pirfenidone treatment with depletion of macrophages. (**F**–**I**) Histologic quantification of edema, inflammation, and necrosis in pancreas is shown, which concurs with our findings. (**J**–**O**) mRNA levels of proinflammatory markers TNF-α, IL-6, IFN-γ, IL-23, IL-12α,and NLRP3 did not show a significant change with pirfenidone treatment with depletion of macrophages. (**P**) Serum HMGB1 levels are not significantly different at 144 hours with macrophage depletion. Pirf., pirfenidone; Clod., liposomal clodronate. *n* =7 in each group. Data represent mean ± SEM. *P < 0.05 by Mann-Whitney *U* test.

To further substantiate our finding that the mechanism of pirfenidone action is through increased IL-10 release from macrophages, which paves the way for alleviation of local and systemic injury in pancreatitis, we demonstrate that the salutary effects of pirfenidone are abrogated either in the absence of IL-10 (IL-10–KO mice) or reparative M2 macrophages (by depleting macrophages using clodronate liposomes in later phases of pancreatitis). As was seen previously, pirfenidone improves pancreas and lung histology at 144 hours (Supplemental Figure 6). However, in IL-10–KO mice, the recovery in pancreas and lung histology (Supplemental Figure 10, A–F) or the decrease in mRNA levels of proinflammatory markers (Supplemental Figure 10, G–L) with pirfenidone treatment was not seen at 144 hours. To further demonstrate the pivotal role played by macrophages in the mechanism of pirfenidone action, we depleted macrophages starting at 96 hours — i.e., 24 hours after the peak of injury — using i.p. injections of liposomal clodronate. The schematic of the experiment is shown in Figure 11A. Macrophage depletion was confirmed using flow cytometry in pancreas and spleen (Figure 11, B and C), and the gating strategy for the same is shown in Supplemental Figure 11, G and H. The effects of pirfenidone were abrogated in absence of macrophages during the recovery phases of pancreatitis, as evidenced by lack of improvement in pancreatic histology (Figure 11D), lung histology (Figure 11E), and histology scores (Figure 11, F–I). The message levels of proinflammatory cytokines also did not show a decrease (Figure 11, J–O). Serum HMGB1 levels also followed the same pattern, with no decrease with pirfenidone treatment in the absence of macrophages (Figure 11P). The H&E pictures of pancreas and lungs with lower magnification (50×) of these studies are given in Supplemental Figure 11, A–F.

## Discussion

There is no specific therapy for AP. In the current manuscript, we have demonstrated that pirfenidone, an antifibrotic, antioxidant, and antiinflammatory drug that is FDA approved for the treatment of IPF, is effective in decreasing the severity of AP in multiple animal models of this disease. Our study is unique in that the pirfenidone treatment was delivered in a therapeutic setting (i.e., after initiation of the pancreatic injury), simulating treatment of patients who present to medical attention much after the pancreatic injury is initiated. Mechanistically, we demonstrate that pirfenidone “quietens” inflammation during AP by acting at multiple levels. Our results suggest that pirfenidone reduces cytokine output from acinar cells, as well as macrophages, and disrupts acinar cell–macrophage crosstalk. Our results also suggest that pirfenidone augments IL-10–driven antiinflammatory pathway in macrophages, and this contributes significantly to its antiinflammatory action in AP. Since pirfenidone is already FDA approved and has been found to be safe for use in humans, these results will form the basis of a clinical trial evaluating the efficacy of pirfenidone for the treatment of AP.

Therapeutic agents for AP should be evaluated in models with established disease (16). To simulate clinically relevant situations, in our experiments, we have administered pirfenidone in a therapeutic setting. For instance, we have administered pirfenidone after 8 injections of caerulein in a 2-day model and after 36 or 72 hours of initiation of L-arginine pancreatitis. That pirfenidone is able to reduce inflammation and injury in these models, even when administered after initiation of injury, is very promising. Given that pirfenidone is already shown to be safe in humans, a clinical trial of pirfenidone for patients at high risk of severe disease could be expeditiously initiated.

While we and others (17–20) have demonstrated that pirfenidone has definite antiinflammatory activity, its mechanism of action is unclear. The various mechanisms proposed for its action include inhibition of NLRP3 inflammasome activation (19), anti–TNF-α activity (21), reduction of macrophage infiltration (18, 22), and antioxidative effect (19, 23). A preliminary report by El-Kashef et al. (24) has suggested that prophylactically administered pirfenidone decreases severity of L-arginine AP by reducing apoptosis. We have performed detailed evaluation of the mechanism by which therapeutically administered pirfenidone modulates severity of AP. Our study demonstrates that pirfenidone can modulate inflammation during AP at various levels. Our in vitro and in vivo studies suggest that, while pirfenidone does not affect trypsin activation, it does affect NF-κB activation in the acinar cells. Furthermore, pirfenidone also affects the extraacinar early event during AP, the acinar cell–macrophage crosstalk, which is known to magnify the local inflammation to systemic level. While pirfenidone treatment, when administered prophylactically, affects these early events, it is unlikely that the ability of pirfenidone to affect AP when administered in therapeutic fashion is due to its impact on these early events. Our data suggest that therapeutically administered pirfenidone reduces inflammation both at local and systemic levels. It changes the phenotype of the inflammatory cells, as is evident from the increase in secretion of antiinflammatory cytokine IL-10 from macrophages preceding changes in histology or a decrease in the message levels of proinflammatory cytokines and proinflammatory cytokine secretion from immune cells. Subsequently, pirfenidone affects the neutrophilic and macrophage infiltration into the pancreas at later stages. Furthermore, our in vitro studies show that pirfenidone reprograms macrophages to reparative M2 phenotype in M1 polarizing conditions and increases the secretion of IL-10. Additionally, pirfenidone also reduces M1 markers at protein levels. M2 macrophages predominate in the later phases of pancreatitis and play a crucial role in promoting repair during recovery from pancreatitis. Given the pivotal role of macrophages in orchestrating inflammation and repair in AP, we need to modulate and not completely abrogate this major player (25).

The effect of pirfenidone on splenocytes isolated at the peak of injury and cultured with or without the drug show a reduction in Ly6C^+^TNF-α^+^ and Ly6C^+^MHCII^+^ cells with pirfenidone treatment. Interestingly, Perides et al. (26) have shown that the Ly-6C^hi^ monocyte subset (which may correspond to M1 macrophages) increased the AP severity by producing TNF-α, and their depletion (using diphtheria toxin–inducible Ly-6C^hi^ monocyte depletion) prior to AP induction in CD11b-DTR mice reduced AP severity. This suggests that at least some, if not all, of the effect of therapeutically administered pirfenidone on AP severity is mediated by a reduction of inflammatory phenotype of macrophages. However, the mechanism by which pirfenidone affects the inflammatory phenotype of macrophages needs to be elucidated.

As mentioned above, besides attenuating the inflammatory phenotype of immune cells, therapeutic pirfenidone increases the synthesis and secretion of IL-10, an antiinflammatory cytokine, from macrophages. IL-10 is a negative-feedback regulator cytokine that is secreted by, as well as inhibits activation of, macrophages and DCs (27). It inhibits production of various inflammatory cytokines like IL-1, TNF-α, and IL-12 from macrophages (27, 28). IL-10 also suppresses T cell activation by inhibiting the expression of costimulators and MHCII on macrophages and DCs (27). It is produced by many immune cells, including activated macrophages, DCs, Tregs, Th1 cells, Th2 cells, and regulatory B cells (27, 28). Based on our data and this literature, we hypothesized that pirfenidone is inhibiting proinflammatory phenotype through increased IL-10 signaling in macrophages. In fact, our results support this hypothesis. Pirfenidone is unable to improve the severity of AP in the absence of IL-10 or depletion of macrophages in the later phases of pancreatitis. Furthermore, in the absence of IL-10, pirfenidone is unable to inhibit the proinflammatory phenotype of immune cells, again suggesting that IL-10 is critical for the effects of pirfenidone in AP. Our hypothesis is also supported by published data, however limited, on the role of IL-10 in AP. Prophylactic administration of recombinant IL-10 in the caerulein–1-day model of AP has been shown to significantly reduce serum amylase and lipase levels with moderate reduction in edema and inflammation, as well as with remarkable improvement in necrosis, on histology (29). IL-10–KO mice have more severe pancreatitis and reduced acinar proliferation in the caerulein–1-day model of AP. (30) In congruence with our results, pirfenidone has been shown to significantly suppress TNF-α, IL-12, and IFN-γ but remarkably increase IL-10 levels in serum after LPS-galactosamine challenge, thereby protecting mice from endotoxic shock in a dose- and time-dependent manner. Intriguingly, it has been shown by us (unpublished data) that pirfenidone, being an anti–TGF-β agent, reverses fibrosis in chronic pancreatitis by immune modulation (31, 32). Furthermore, the mechanism by which pirfenidone affects IL-10 signaling in macrophages is not known and will require in-depth molecular studies, which we will undertake in the near future.

In summary, therapeutic approaches that modulate the leukocyte phenotype to one with antiinflammatory properties offer plausible treatment strategy for AP (16). Pirfenidone treatment ameliorates the severity of pancreatic and systemic injury in AP in various mouse models, not only in a prophylactic setting, but also when administered therapeutically in models of well-established disease. Pirfenidone interrupts the inflammatory cascade at multiple levels through immune modulation and cytokine modulation, with IL-10 release from M2 macrophages being crucial for its effect. As pirfenidone is already FDA approved for IPF, our study can become the basis of its clinical evaluation as a treatment strategy for patients with moderate to severe AP.

## Methods

### Experimental mice.

All in vivo experiments were conducted using 6- to 8-week-old male C57BL/6J mice weighing 22.5–27 g. They were either in-bred in our facility or purchased from the Jackson Laboratory. IL-10 reporter mouse (C57BL/6J background) (14) breeder pairs were donated by Craig L. Maynard’s laboratory in University of Alabama at Birmingham and in-bred in our laboratory. IL-10–KO mice (C57BL/6J background) breeder pairs were purchased from the Jackson Laboratory and in-bred in our facility. Male mice were used for experiments when they were 6–8 weeks old. All in vitro experiments were conducted using 6- to 8-week-old female C57BL/6J mice weighing 22–27 g. Mice were kept in environment-controlled microisolators under specific pathogen–free conditions with a 12-hour light/dark cycle and an ambient temperature of 23°C ± 2°C. Mice were given standard laboratory chow (LabDiet) as feed and provided water ad libitum. Mice purchased from the vendors were acclimatized to this environment for at least 1 week prior to starting experiments.

### Experimental AP.

All in vivo experiments were performed 3 times by 3 independent investigators to ensure reproducibility of data. AP was induced by either repeated hourly injections of a cholecystokinin analog, caerulein (Bachem, catalog 403045) or 2 injections of L-arginine (Sigma-Aldrich, catalog 11039) given an hour apart i.p. to C57BL/6J mice. Caerulein-induced AP was either a 1-day or a more severe 2-day model with caerulein (50 μg/kg, i.p.) administered 8 times hourly for 1 or 2 days, respectively. Pirfenidone (MedChemExpress, catalog HY-B0673) (400 mg/kg) was administered by oral gavage after dissolving it in warm normal saline. For the 1-day model of caerulein-induced AP, pirfenidone was administered in a prophylactic manner. In the prophylactic treatment group, pirfenidone (400 mg/kg, oral gavage) was given 12 hours and 1 hour before the first injection of caerulein, and a third dose was given with the fifth injection of caerulein. All animals were euthanized 1 hour after the last injection of caerulein by CO_2_ asphyxiation. Plasma and tissue samples of pancreas as well as lungs were harvested for subsequent analyses. In the therapeutic 2-day model of caerulein AP, pirfenidone gavages were given at 9 hours, 24 hours, and 48 hours after the first injection of caerulein. Mice were sacrificed at 56 hours after the start of the model, and appropriate samples were collected. A group of mice (*n* = 8) was sacrificed 1 hour after the last injection of caerulein for measuring amylase levels and evaluating pancreatic and lung wet/dry weight ratio (a measure of edema) as it decreases to control levels in AP mice at 56 hours. The wet/dry weight ratio was evaluated by weighing the organ before and after incubating in an incubator at 56°C for 24 hours and calculating the ratio.

The L-arginine model of AP was induced using the protocol previously described by our group (33), with a few modifications. AP was induced by administration of 2 i.p. injections of L-arginine (4.5 g/kg/injection) 1 hour apart. To reduce mortality, fluid resuscitation with normal saline bolus (100 μL/g/dose up to a maximum of 2.5 mL, given s.c.) was administered twice at 1-hour intervals after the last injection of L-arginine. Pirfenidone (400 mg/kg) was given at 36, 48, and 60 hours after the first injection of L-arginine — i.e., after initiation of injury. Mice were euthanized at 72 hours after the first injection of L-arginine, and samples were collected. In the well-established L-arginine model with pirfenidone started therapeutically, the presence of AP was confirmed using a more-than 3-fold increase in amylase levels in plasma obtained by retro-orbital bleeding using heparinized capillary tubes (Kimble Chase, catalog 505) after giving isoflurane anesthesia at 72 hours (the peak of injury). The AP mice were randomized to control and treatment groups. Pirfenidone (400 mg/kg) was administered in the treatment group at 72- (the peak of injury), 96-, and 120-hour time points from the time of the first injection of L-arginine. Mice in each group were euthanized at 72 (pretreatment), 96, 120, and 144 hours. The samples were harvested for subsequent analyses as previously described. A similar experiment was done exclusively for flow cytometric analysis of immune cells. In the IL-10–depletion experiment — in the same well-established model of L-arginine AP, as described above (with pirfenidone started at 72 hours) — anti–mouse IL-10 neutralizing antibody (0.25 mg, JES5-2A5, Bio X Cell, catalog BE0049) or isotype control antibody (Bio X Cell, catalog BE0088) was given i.p. at 72-, 96-, and 120-hour time points, and mice were sacrificed at 4 hours after the last injection; samples were collected for analyses. To further dissect the mechanism, mice were sacrificed at 110 hours when the histology did not show any change with treatment in well established L-arginine model. For immune characterization at 110 hours, IL-10 reporter mice (10BiT mice on C57BL/6J background) (14) were used. The macrophage panel was stained for Thy1.1 (a surrogate surface marker for IL-10) in IL-10 reporter mice (14). For macrophage depletion, mice were i.p. given 200 μL of liposomal clodronate or control liposomes (FormuMax Scientific, catalogs F70101C-N and F70101-N) at 96 and 120 hours.

### Tissue collection and morphological examination.

At the time of sacrifice, pancreatic and lung tissue samples were snap frozen in liquid nitrogen and stored at –80°C for subsequent protein and mRNA analysis. For histologic evaluation, pancreatic and lung tissues were fixed in 10% neutral phosphate buffered formalin for 24–48 hours. Pancreatic and lung tissue sections (4 μm) were then stained with H&E and evaluated by a morphologist blinded to the groups as well as the results. Images were acquired on a light microscope. Ten microscopic pancreatic fields (100×) were randomly selected per mouse by an investigator blinded to results, and pancreatic edema, necrosis, and inflammatory infiltrate were quantified by histology scoring in a blinded fashion as previously described (34). The histology scoring is as follows: edema (0, absent; 1, focally increased between lobules; 2, diffusely increased between lobules; 3, tense acini, widely separated lobules; 4, gross lobular separation); necrosis (0, absent; 1, obvious edema and minimal necrosis; 2, focal parenchymal necrosis [<20%]; 3, diffuse loss of lobules [20%–50%]; 4, severe loss of lobules [>50%]); and inflammation (0, absent; 1, around ductal margins; 2, in parenchyma [<50% of lobules]; 3, in parenchyma [51%–75% of lobules]; 4, massive collections).

### IHC.

Immunostaining for coronin 1A on pancreatic and lung tissue sections (4 μm) was done using coronin antibody (1:1000) (Bethyl Laboratories, catalog A300-930A), as previously described by our group (35). Representative images were acquired on a Leica microscope.

### ELISA, myeloperoxidase activity, and serum amylase.

ELISA was performed on serum samples and pancreatic tissue homogenates from in vivo experiments and cell culture supernatants from in vitro coculture experiments. Mouse IL-6, TNF-α, and HMGB1 ELISA kits were purchased from MilliporeSigma (catalog RAB0308), Invitrogen (catalog 88-7324-22), and Chondrex (catalog 6010), respectively. Mouse CRP ELISA kit and mouse IL-10 ELISA kit were purchased from R&D Systems (catalogs MCRP00 and M1000B, respectively). The assays were performed according to the manufacturer’s instructions.

Myeloperoxidase activity, a marker of neutrophilic infiltration and inflammation, was measured colorimetrically in both the pancreas and lung tissues after tissue homogenization, as previously described by our group (36). Serum amylase was measured using amylase assay reagent according to manufacturer’s instructions (Sekisui, catalog 80-5383-00).

### Flow cytometry.

Single-cell suspensions of pancreata were made after collagenase digestion with collagenase type 4 (MP Biomedicals, catalog 195110). The cells were then treated with Golgi Plug (BD Biosciences, catalog 51-2301KZ), PMA (100 ng/mL, Stem Cell Technologies, catalog 74042), and ionomycin (500 ng/mL, Stem Cell Technologies, catalog 73722) for 4 hours at 37°C and subsequently fixed overnight. For surface staining for macrophage/neutrophil markers, cells were stained with the following fluorochrome conjugated antibodies from BioLegend in the macrophage/neutrophil panel: CD45-APC (catalog 103112), F4/80-BV421 (catalog 123132), CD11b-PerCP-Cy5.5 (catalog 101228), MHCII-AF 700 (catalog 107622), CD206-AF488 (catalog 141710), Ly6C-PE/Cy7 (catalog 128018), Ly6G-APC/Cy7 (catalog 127674), Thy 1.1- PE Dazzle (catalog 202541), and Ly6G/Ly6C (Gr-1)-PE (catalog 108408). For surface staining of the T cell/B cell/NK cell panel, the following fluorochrome conjugated antibodies from BioLegend were used: CD3-PE Dazzle (catalog 100348), CD4-PE Cy7 (catalog 100528), CD8-AF-647 (catalog 100724), CD19-PerCP5.5 (catalog 115534), CD49b-FITC (catalog 108906), and CD25-AF-700 (catalog 102024).

Permeabilization buffers for intracellular staining were purchased from Invitrogen (eBioscience, catalogs 00-5123-43, 00-5223-56, and 00-833356). For intracellular staining after permeabilization, fluorochrome conjugated antibodies from BioLegend were used. TNF-α– BV650 (catalog 506333), IL-4-BV711 (catalog 504133), and IL-10-PE Dazzle (catalog 505034) for the macrophage panel and IL-10-APC/Cy7 (catalog 505036) for the T cell/B cell/NK cell panel. IL-17-BV-421 (catalog 506926) and FOXP3-PE (catalog 126404) were used for T cells. Cells were then fixed in fixation buffer (BD Pharmingen, catalog 505034) and acquired using LSR Fortessa II (BD Biosciences) at University of Miami or BD FACSymphony A5 at University of Alabama at Birmingham. The resulting data were analyzed using FlowJo software (Tree Star Inc.).

### RNA isolation, cDNA preparation, and quantitative PCR (qPCR).

Pancreatic tissue was homogenized using MagNa Lyser tubes (Roche, catalog 03358941001) in TRIzol reagent (Thermo Fisher Scientific, catalog 15596026) and RNA was isolated using Qiagen RNEasy Plus Mini kit (Qiagen, catalog 74134) according to manufacturer protocol. cDNA was isolated using high-capacity cDNA Reverse Transcription Kit (Applied Biosystems, catalog 4368814). The primer sequences used are provided in the Table 1. Quantitative real-time detection of cDNA was performed using Light Cycler 480 II (Roche) after adding the primer mixes and SYBR green (LightCycler 480 SYBR Green I Master, catalog 04887352001) in triplicate according to the manufacturer recommendations.

### Western blotting.

Pancreatic tissue was homogenized in RIPA buffer containing protease and phosphatase inhibitors. Protein quantification was done by the BCA method using Pierce BCA Protein Assay Reagent (Thermo Fisher Scientific, catalogs 23228 and 23224). In total, 40 μg of protein was loaded in each well, separated by polyacrylamide gel electrophoresis, and then transferred to nitrocellulose membrane. The gels used were Mini-PROTEAN TGX Stain-free gels (Bio-Rad, catalog 456-8094). Membrane blocking was performed in 5% skimmed milk, followed by incubation with primary antibody (IκBα [Cell Signaling Technology, catalog 9242] and GAPDH [MilliporeSigma, catalog MAB374]). Horseradish peroxidase–conjugated secondary antibodies (Anti-mouse IgG, HRP-linked Antibody, catalog 7076 and Anti-rabbit IgG, HRP-linked Antibody, catalog 7074; both from Cell Signaling Technology) were used to be detected by chemiluminescence.

### Multianalyte flow assay for cytokines.

Cytokine levels in mice serum were quantified using Legendplex Multi-Analyte Flow Assay Kit from BioLegend (catalog 740150), including TNF-α and IL-17A.

### Trypsin activity.

For all in vitro studies, samples were always plated in 2–3 technical replicates and done at least 2–3 times (biological replicates). For in vitro trypsin assay, acinar cells were isolated and plated in 6-well plates in HEPES buffer. In the treatment groups, cells were pretreated with pirfenidone (0.5 mg/mL) 30 minutes prior to adding supramaximal carbachol (1 mM). Cells were then collected after 1 hour. Acinar cells were homogenized in 3-(N-morpholino)-propane sulfonic acid buffer, pH 6.5 (250 mmol/L sucrose, 5 mmol/L MOPS, 1 mmol/L MgSO4), using a sonicator. Homogenates were centrifuged (835*g* for 5 minutes) at 4°C, and supernatants were used for trypsin enzyme activity measurement. Trypsin activity was determined ﬂuorometrically using Boc-Glu-Ala-Arg-MCA as substrate as described earlier (37).

### Western blotting for IκBα degradation.

C57BL/6J mice were administered 1 injection of caerulein (50 μg/kg, i.p.) and euthanized after 1 hour in the caerulein-only group. Mice were pretreated with pirfenidone oral gavage (400 mg/kg) 30 minutes before giving caerulein in the pirfenidone group (*n* = 3 in each group). Pancreatic tissue was harvested after 1 hour of giving caerulein, and Western blotting for IκBα degradation was done as described previously.

### EMSA.

Acinar cells were isolated as previously described (38) and cultured in 6-well plates in 1 mL of media (DMEM without phenol red [Thermo Fisher Scientific, catalog 21063029] with 0.2% BSA, 1% penicillin/streptomycin, and soya bean trypsin inhibitor) with or without supramaximal caerulein (100 nM) and with or without pirfenidone (0.5 mg/mL) in duplicate for 1 or 3 hours. At the end of incubation, nuclear fraction was extracted using Abcam Nuclear Extraction Kit (catalog ab113474) using manufacturer-recommended protocol. The EMSA was done using Light Shift Chemiluminescent EMSA kit (Thermo Fisher Scientific, catalog 20148) according to manufacturer-recommended protocol and controls. The NF-κB p65 DNA binding sequence (from Integrated DNA Technologies) was as follows: forward sequence 5′ AGT TGA GGG GAC TTT CCC AGG C 3′ and reverse sequence 5′ GCC TGG GAA AGT CCC CTC AAC T 3′. In total, 3 μg of protein was loaded in each well, and the exposure time to x-ray film was 20–30 seconds.

### Acinar cell culture.

Acinar cells were isolated as previously described (38) and cultured in 6-well plates in 1 mL of media (DMEM without phenol red (Thermo Fisher Scientific, catalog 21063029) with 0.2% BSA, 1% penicillin/streptomycin, and soya bean trypsin inhibitor). The experimental wells with acinar cells were pretreated with pirfenidone (0.5 mg/mL) for 30 minutes prior to adding supramaximal caerulein. ELISA was used to quantitate TNF-α and IL-6 in the supernatant collected after 4 hours of caerulein treatment.

### Acinar cell and macrophage coculture.

Murine BM cells were isolated and transformed into macrophages (BM-derived macrophages [BMDMs]) by adding recombinant murine M-CSF (20 ng/mL) (Peprotech, catalog 315-02) and culturing it for 7 days in DMEM/F-12 with FBS (10%) and 1% penicillin/streptomycin according to previously described protocol (39). Their conversion to macrophages was confirmed by immunofluorescence staining for F4/80^+^ cells (Abcam, catalog ab6640) and through flow cytometry after staining for F4/80^+^ cells (BioLegend F4/80-BV421, catalog 123132). Macrophages were detached from culture plates using TrypLE Express Enzyme (Thermo Fisher Scientific, catalog 12604013) cell dissociation reagent and plated in 6-well plates (250,000 cells/well) in triplicate. They were kept overnight for reattachment with subsequent media change the following morning. These cells were grown for another 3 days prior to the coculture experiment. Three different methods were used to assess acinar-macrophage crosstalk:

In the first method, acinar cells were isolated from mice as previously described (38) and cultured in transwells (Cell culture insert, Falcon, catalog 353090) (approximately 120 mg/well) positioned above macrophages in a total of 1.5 mL of media. These cells were then treated with supramaximal dose of caerulein (100 nM) and cocultured with macrophages for 4 hours in acinar cell culture media (DMEM without phenol red [Thermo Fisher Scientific, catalog 21063029] with 0.2%BSA, 1% penicillin/streptomycin, and soya bean trypsin inhibitor) with or without pirfenidone (0.5 mg/mL) pretreatment for 30 minutes before addition of caerulein. TNF-α and IL-6 were measured in the supernatant by ELISA.

In the second method, isolated acinar cells were treated with a supramaximal dose of caerulein with or without pirfenidone (0.5 mg/mL) and incubated in Hepes 1× buffer for 1 hour. The acinar cells were then washed 3 times with acinar cell culture media to wash any residual caerulein or pirfenidone, and they were then cocultured with macrophages in transwells for 3 hours and analyzed as described in method 1.

In the third method, acinar cells were isolated and treated with a supramaximal dose of caerulein with or without pirfenidone (0.5 mg/mL) and incubated in acinar cell culture media for 3 hours. Subsequently, the supernatant of the respective groups was transferred to macrophages in 6-well plates and incubated for a further 3 hours, analyzed as described in method 1. The mRNA was isolated from macrophages and analyzed using qPCR for TNF-α and IL-6.

MTT (3-[4,5-dimethylthiazol-2-yl]-2,5-diphenyltetrazolium bromide) cell proliferation assay kit was used to assess macrophage viability at 24- and 72-hour incubation times (Cayman Chemical, item no. 10009365).

### Splenocytes culture.

Spleens were harvested from C57BL/6J mice with L-arginine AP at peak of injury at 72 hours (*n* = 6 each) or with caerulein AP (*n* = 3 each) 1 hour after the last injection of caerulein. Single-cell suspension of splenocytes were made and cultured in 6-well plates in duplicate with or without pirfenidone (0.5 mg/mL) in 2 mL RPMI media with 10%FBS, L-glutamine (200 mM), sodium pyruvate 1 mM, and 1% penicillin/streptomycin for 12 hours. Cells were then detached, stained for flow cytometry, and acquired as described above. Ly6C was used as a marker of monocyte/macrophage lineage, as it is a better marker than F4/80 in splenocytes (40).

### Statistics.

Either Kruskal-Wallis test (Dunn’s multiple-comparison est), ordinary 1-way ANOVA, or 2-way ANOVA was used for comparison between multiple groups. Mann-Whitney *U* test (nonparametric test) was used for comparison between 2 groups. A *P* value of less than 0.05 was considered statistically significant. Results are expressed as mean ± SEM (Prism 6; Graph Pad Software).

### Study approval.

All animal experiments were performed after approval of protocol from the IACUCs at University of Miami and University of Alabama at Birmingham.

## Author contributions

EPB, JG, and SI performed experiments; EPB, JG, MT, and SI acquired data; EPB, JG, MT, SI, PS, BG, VS, TJ, PS, AF, and VD analyzed data; HKCJ, UV, BGA, and DBE provided reagents; VD and RKD conceptualized and supervised the project; VD, RKD, and CLM designed the experiments; VD, EPB, and BG wrote the manuscript; EPB, VD, and RKD revised the manuscript; VD and AKS provided resources.

## Supplementary Material

Supplemental data

## Figures and Tables

**Table 1:**
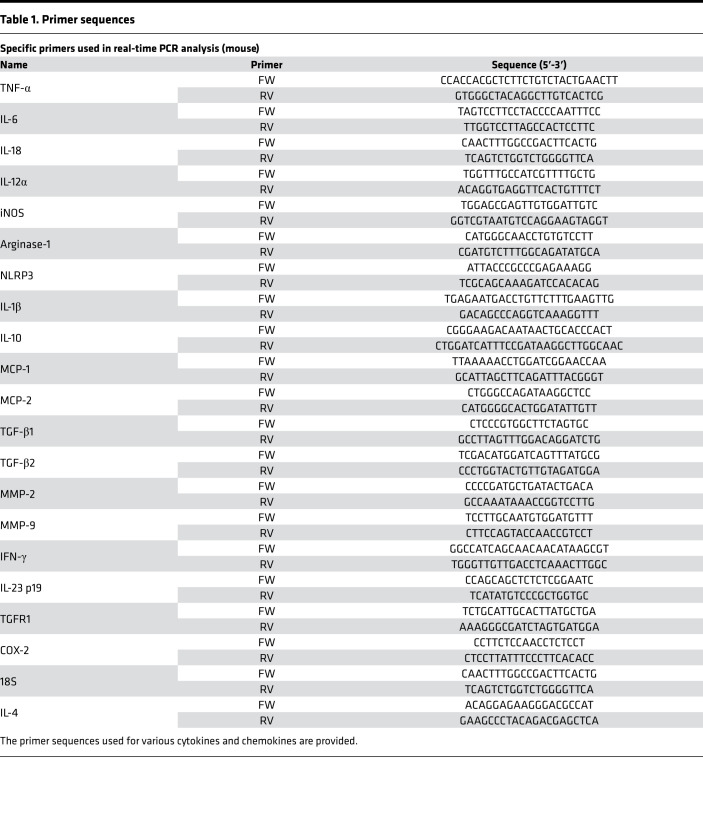
Primer sequences

## Version 1 11/30/2021

In-Press Preview

## Version 2 01/25/2022

Electronic publication
