# Supplementary material for: Pirfenidone increases IL-10 and improves acute pancreatitis in multiple clinically relevant murine models
Source: JCI Insight. 2022 Jan 25;7(2):e141108. doi: 10.1172/jci.insight.141108 (PMC8855813; doi:10.1172/jci.insight.141108)
Supplement: Supplemental data [file jciinsight-7-141108-s197.pdf]

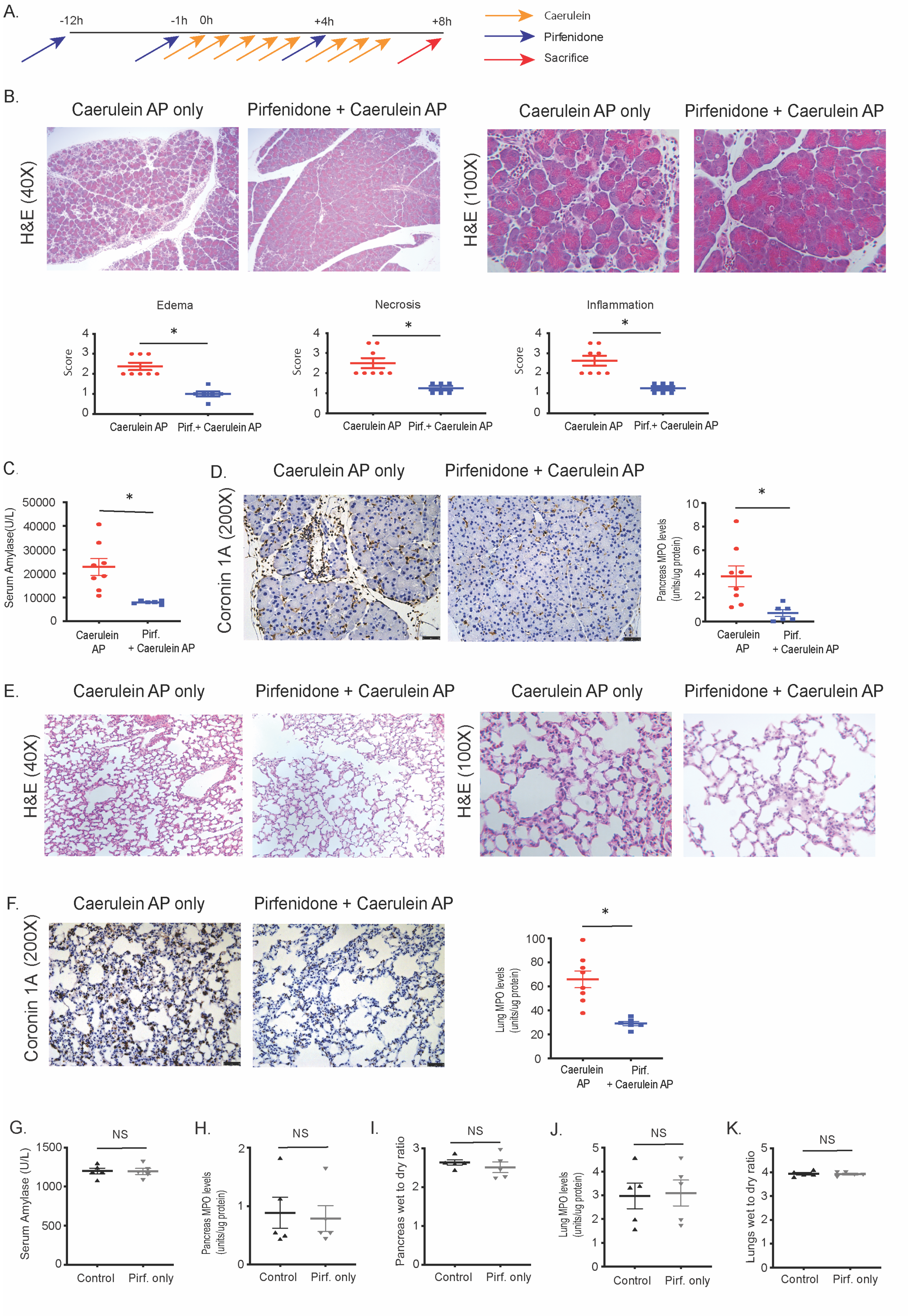

Supplementary figure 1

Supplementary Figure 1: Prophylactically administered pirfenidone reduces local pancreatic injury and lung injury in caerulein 1-day model of acute pancreatitis

(A) Schematic of pirfenidone administration in caerulein 1-day model of acute pancreatitis. (B) Histological analysis of representative H&E sections (40X and 100X) of pancreata from AP only group and pirfenidone treated AP group. Histologic quantification of edema, necrosis and inflammation is also shown. (C) Pirfenidone treatment significantly decreased serum amylase levels. (D) Immunohistochemistry for coronin 1A (200X), which stains leukocytes, shows a decrease in immune infiltration with pirfenidone treatment. Pancreatic MPO which is a marker of neutrophilic infiltration also shows a significant decrease with treatment. (E) Lung H&E (40X and 100X) shows a reduction in injury with pirfenidone treatment. (F) Immunohistochemistry of lung sections for coronin 1A (200X) shows a decrease in immune infiltration with pirfenidone treatment. Lung MPO (myeloperoxidase) also shows a significant decrease with treatment. Pirf.=Pirfenidone; NS=Not significant. n=8 each in AP only group and n=6 each in pirf+AP group. Data represents mean ± SEM. *P < .05 (Mann-Whitney test). [(G) to (K)] Pirfenidone given to control mice (without AP) does not affect (G) serum amylase, (H) pancreas MPO, (I) pancreas wet to dry weight ratio, (J) lung MPO or (K) lung wet to dry weight ratio. n=5 in each group. Data represents mean ± SEM. *P < .05 (Mann-Whitney test).

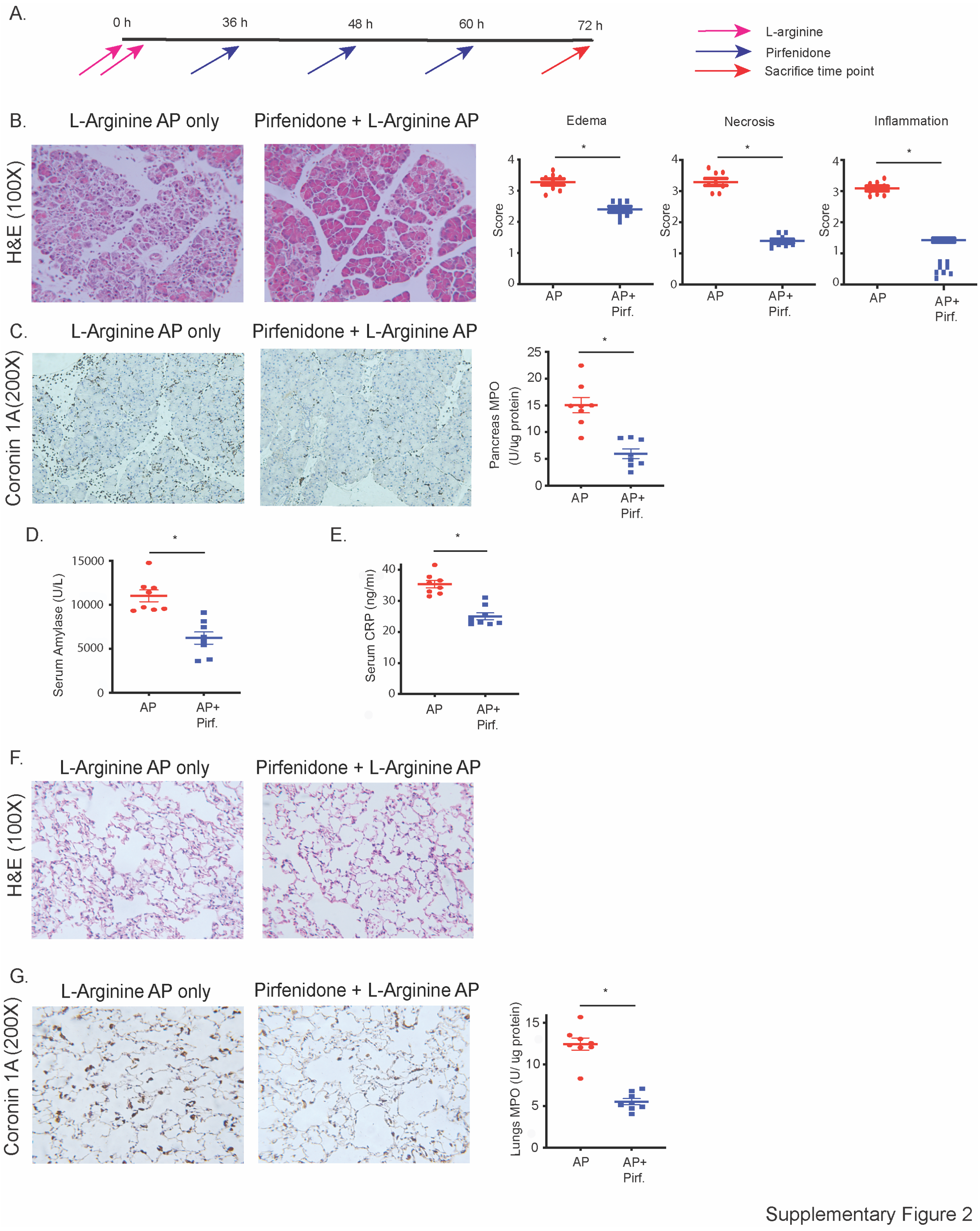

Supplementary Figure 2: Therapeutic pirfenidone reduces local pancreatic injury and lung injury in L-arginine model of acute pancreatitis.

(A) Schematic of pirfenidone administered in a therapeutic manner in L-arginine model of acute pancreatitis (B) Histological analysis of representative H&E sections (100X) of pancreata from AP only group and pirfenidone treated AP group. A decrease in pancreatic edema, necrosis and inflammatory infiltrate can be seen. Histologic quantification of edema, necrosis and inflammation is shown. (C) Immunohistochemistry for coronin 1A (200X), which stains leukocytes, shows a decrease in immune infiltration with pirfenidone treatment. Pancreatic MPO which is a marker of neutrophilic infiltration also shows a significant decrease with treatment. (D) Serum amylase and (E) serum CRP , were significantly reduced with pirfenidone treatment. (F) Lung H&E (100X) shows a reduction in injury with pirfenidone treatment. (G) Immunohistochemistry of lung sections for coronin 1A (200X) shows a decrease in immune infiltration with pirfenidone treatment. Lung MPO (myeloperoxidase) also shows a significant decrease with treatment. Pirf.=Pirfenidone. n =8 in each group. Data represents mean ± SEM *$P < .05$ (Mann Whitney test)

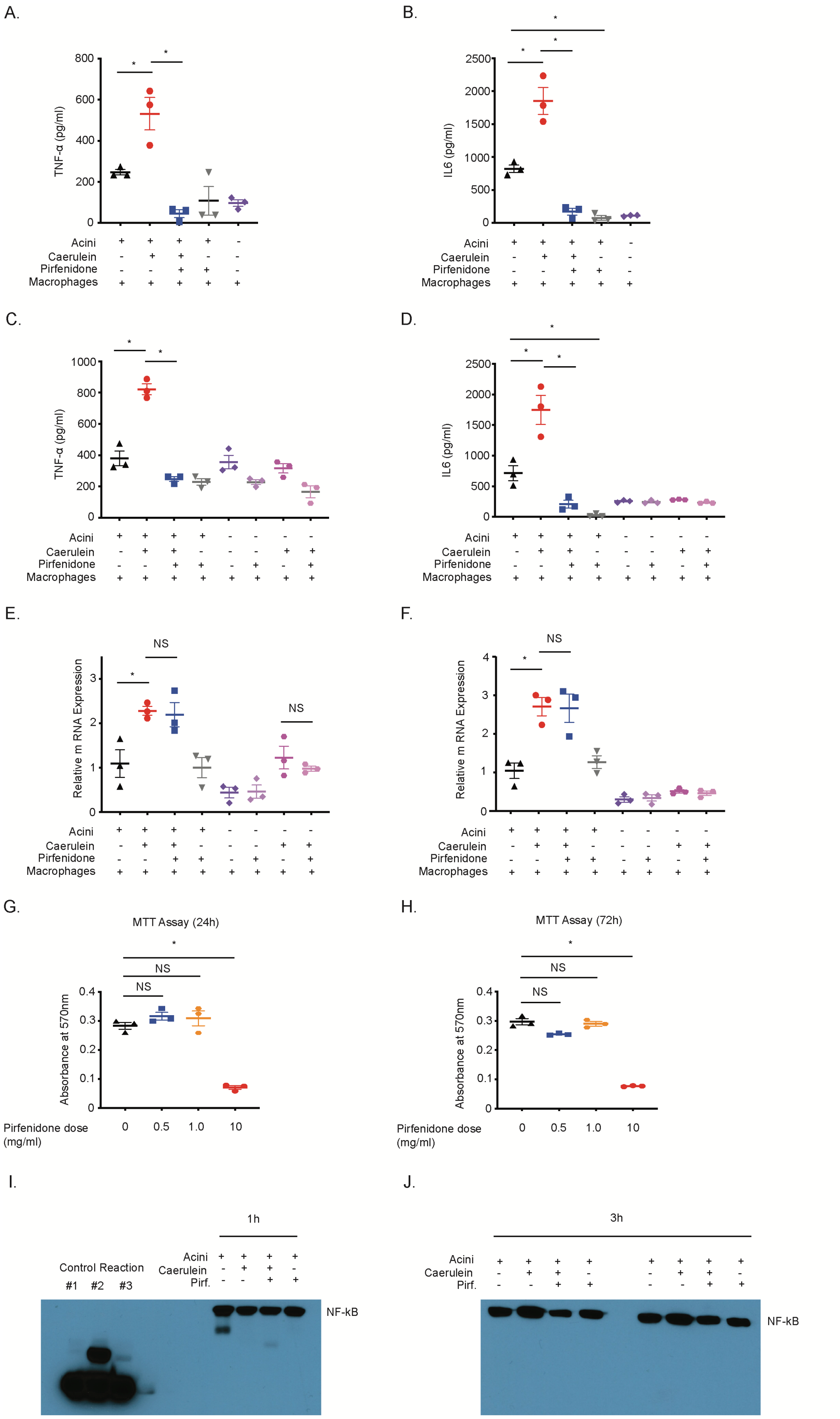

Supplementary Figure 3

Supplementary Figure 3: **Pirfenidone disrupts supra-maximal caerulein induced acinar cell- macrophage crosstalk and reduces NF-kB binding to DNA in-vitro**

(A-D) Pirfenidone significantly reduces TNF-α (A&C) and IL6 (B&D) secretion from macrophages either when co-cultured with acinar cells pre-treated with supra-maximal caerulein for 1h with or without pirfenidone (0.5 mg/ml) and then washed with media before co-culturing with macrophages in trans-wells for 3 hours (A&B) or with transfer of acinar supernatant pre-treated with supramaximal caerulein with or without pirfenidone (0.5 mg/ml) for 3 hours on macrophages, which are cultured alone for another 3 hours (C&D). (E-F) m RNA levels of TNF-α (E) and IL6 (F) from the experiment in (C) &(D) shows that pirfenidone does not affect the m RNA levels of these cytokines. (G) & (H) MTT Assay for macrophage viability at indicated dosages of pirfenidone in-vitro after 24h (G) and 72h (H) of incubation shows that pirf. does not affect the macrophage viability at the doses 0.5 mg/ml (dose used in in-vitro studies) or 1mg/ml. (I)&(J) Effect of pirfenidone (0.5 mg/ml) on the binding of p65 subunit of NF-kB to DNA in acinar cells. Acinar cells were treated with supramaximal caerulein in the presence or absence of pirfenidone (0.5 mg/ml) for (I) 1h or (J) 3h. Nuclear extracts were analyzed by EMSA (electrophoretic mobility shift assay). Pirfenidone reduced NF-kB DNA binding at 3h (I) but not at 1h (J). Experiment was done twice in duplicates with manufacturer recommended controls. Representative images are shown. The unbound DNA is not visible (except in the control reactions) as the binding reactions are too intense. Pirf.=Pirfenidone ; NS=Not significant. n =3 in each group for (A) to (H). n=2 in each group for (I) & (J) Data represents mean± SEM and *P < .05 (Two Way ANOVA for (A) to (F); Ordinary one-way ANOVA for (G) & (H).

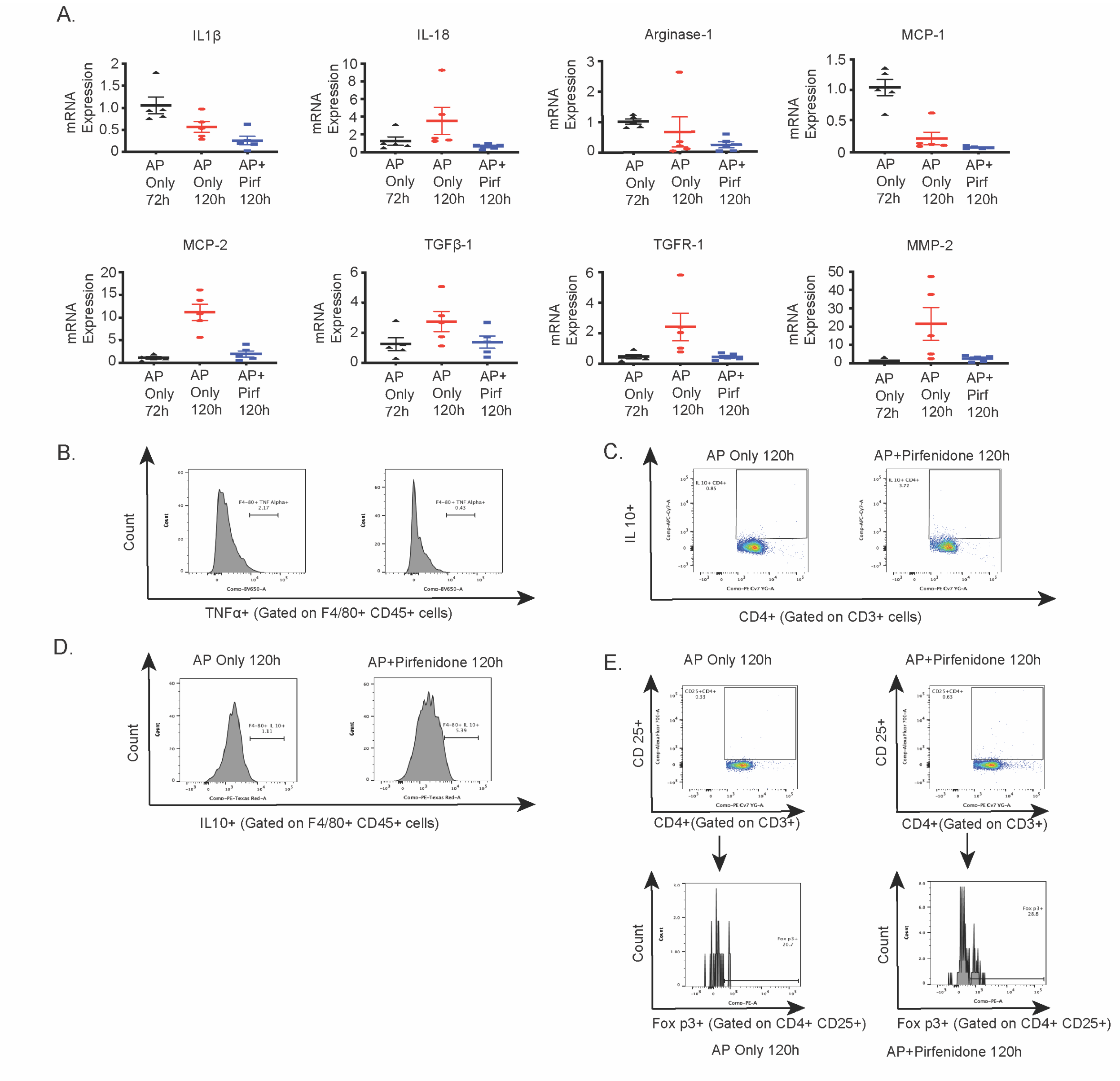

Supplementary Figure 4

Supplementary Figure 4

(A) qPCR analysis of various cytokines and chemokines at 120h time point of L-arginine model of acute pancreatitis that did not show a significant change with pirfenidone treatment. (B-E) The gating strategy for flow analysis at 120h time point of L-arginine AP with pirfenidone started at peak of injury is shown. Pirf.=Pirfenidone. Data represents mean ± SEM and n =5 in each group. *P < .05 [Kruskal-Wallis Test (Dunn's Multiple Comparisons Test)].

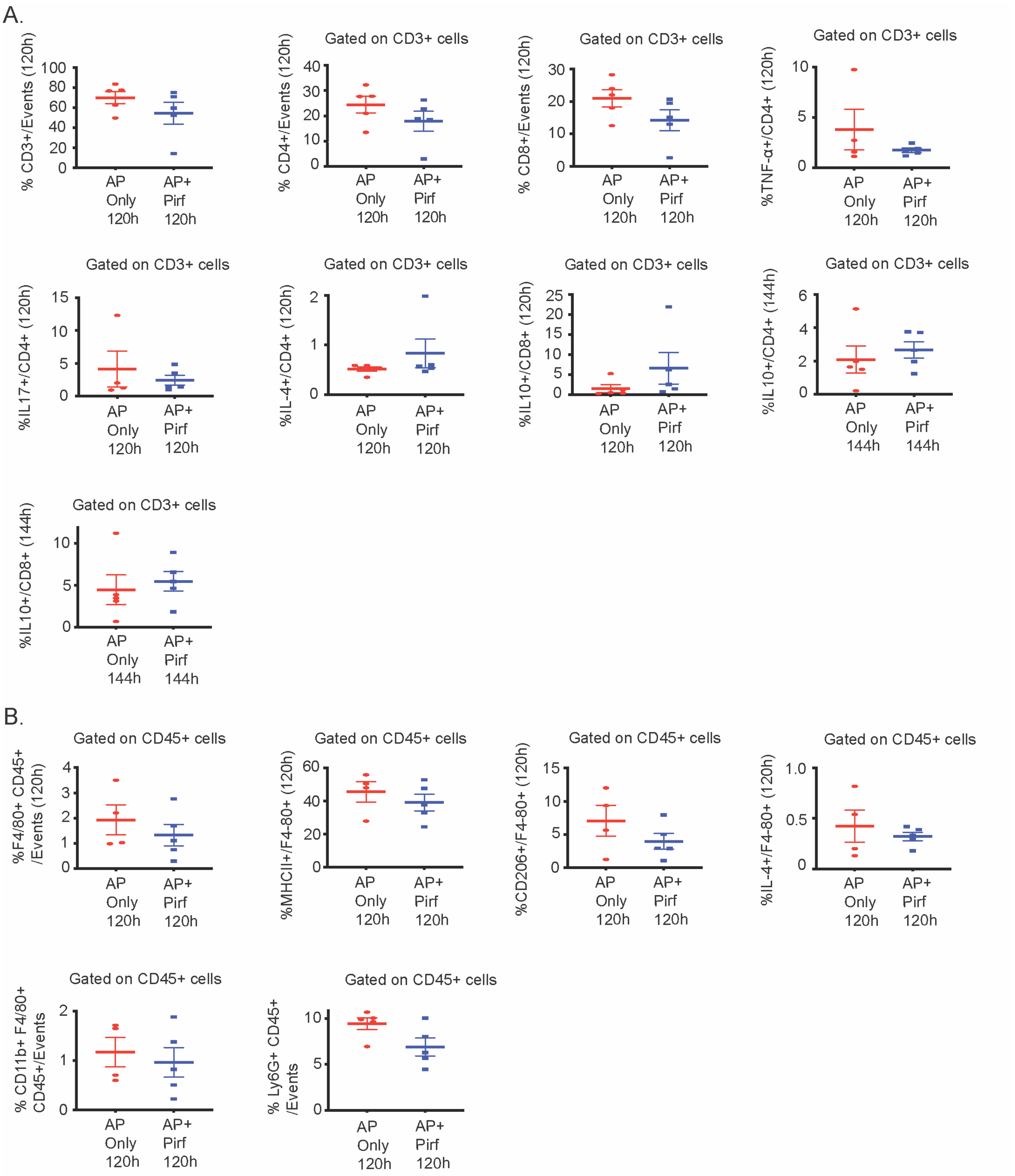

Supplementary Figure 5

Supplementary Figure 5

(A&B) Flow cytometry analysis for T cell panel (A) and macrophage/neutrophil panel (B) at 120h and 144h time points of L-arginine AP which did not show a statistically significant difference

with pirfenidone treatment. Pirf.=Pirfenidone. Data represents mean ± SEM and n =4-5 in each group. *P < .05 (Mann Whitney test).

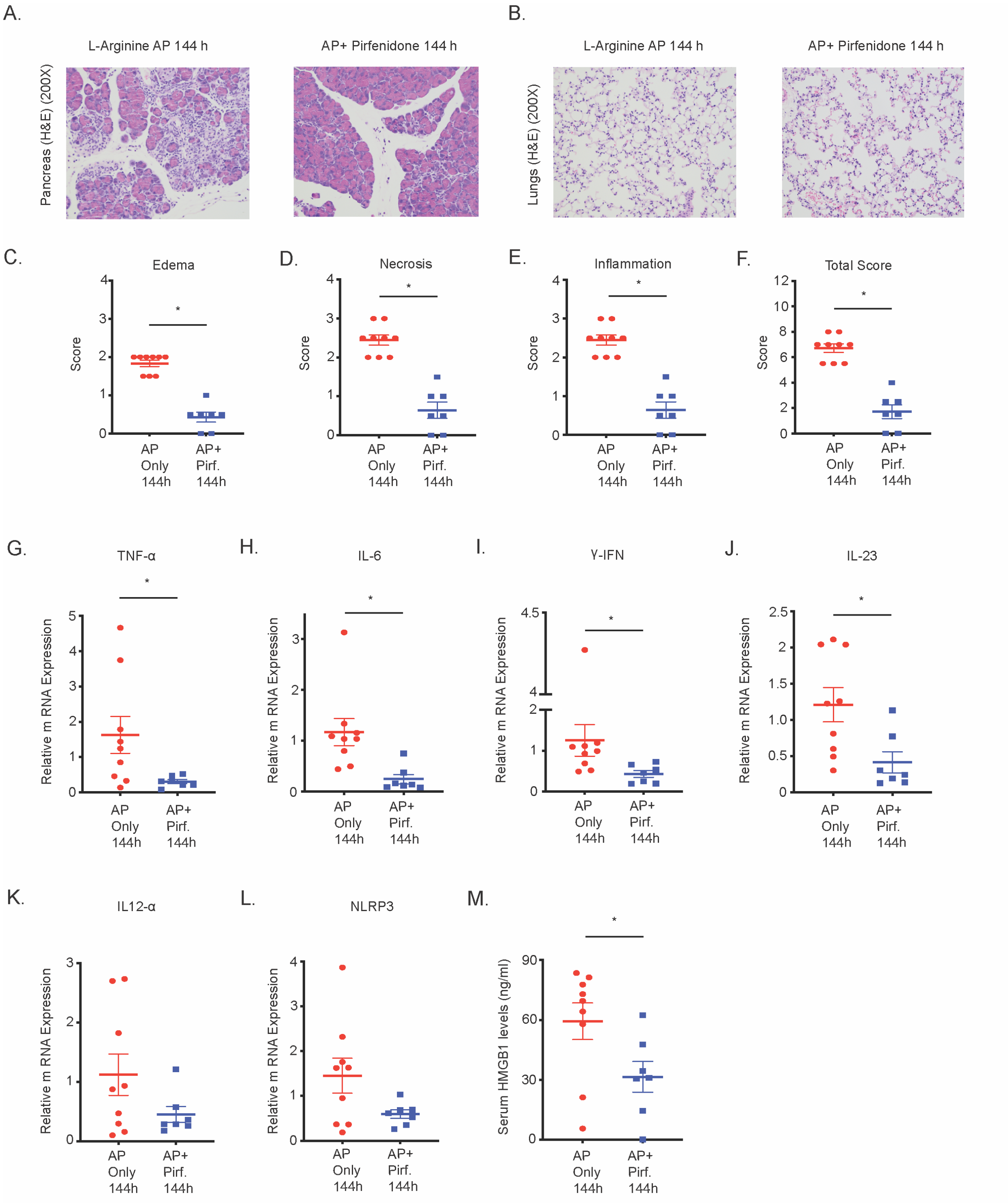

Supplementary Figure 6

Supplementary Figure 6: Therapeutic pirfenidone, when administered at the peak of injury in a well-established L-arginine model of acute pancreatitis, reduces local pancreatic and lung injury at 144h.

(A) Histological analysis of representative H&E sections (200X) of pancreas from AP only group and pirfenidone treated AP group. A decrease in pancreatic edema, necrosis and inflammatory infiltrate can be seen. (B) Lung H&E (200X) shows a reduction in injury with pirfenidone treatment. (C-F) Histologic quantification of edema, necrosis and inflammation is shown which concurs with our conclusion. (G-L) mRNA levels of proinflammatory markers TNF-α, IL-6, IFN-γ and IL-23 showed a significant decrease with pirfenidone treatment, while IL-12 α and NLRP3 showed a decreasing trend. (M) Serum HMGB1 levels are significantly reduced at 144h with treatment. n =9 in AP only group and n=7 in pirfenidone group. Pirf.=Pirfenidone. Data represents mean± SEM. *$P < .05$ (Mann Whitney test).

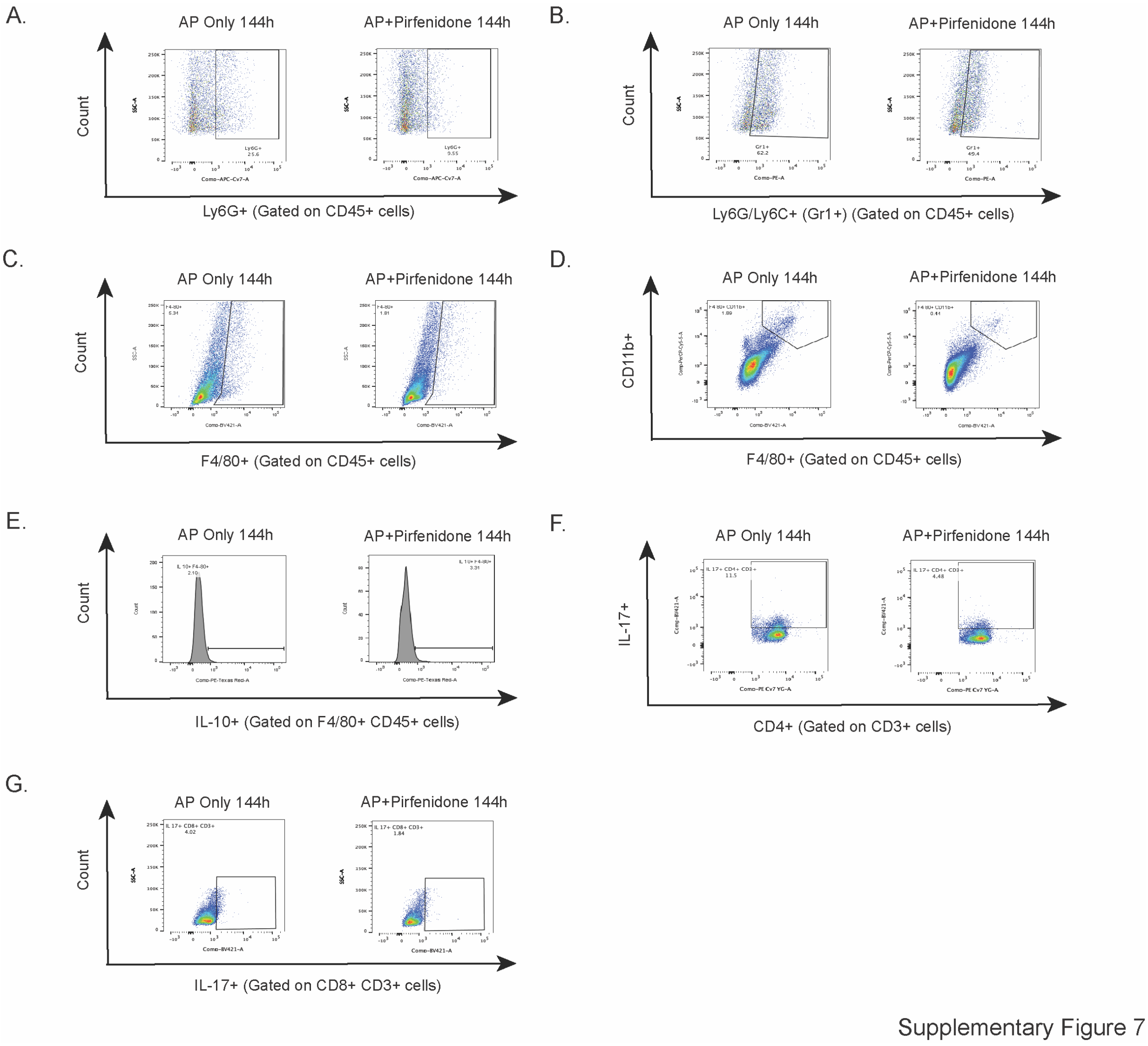

Supplementary Figure 7

(A-G) Gating strategy for flow cytometry analysis of pancreatic immune cells at 144h time point.

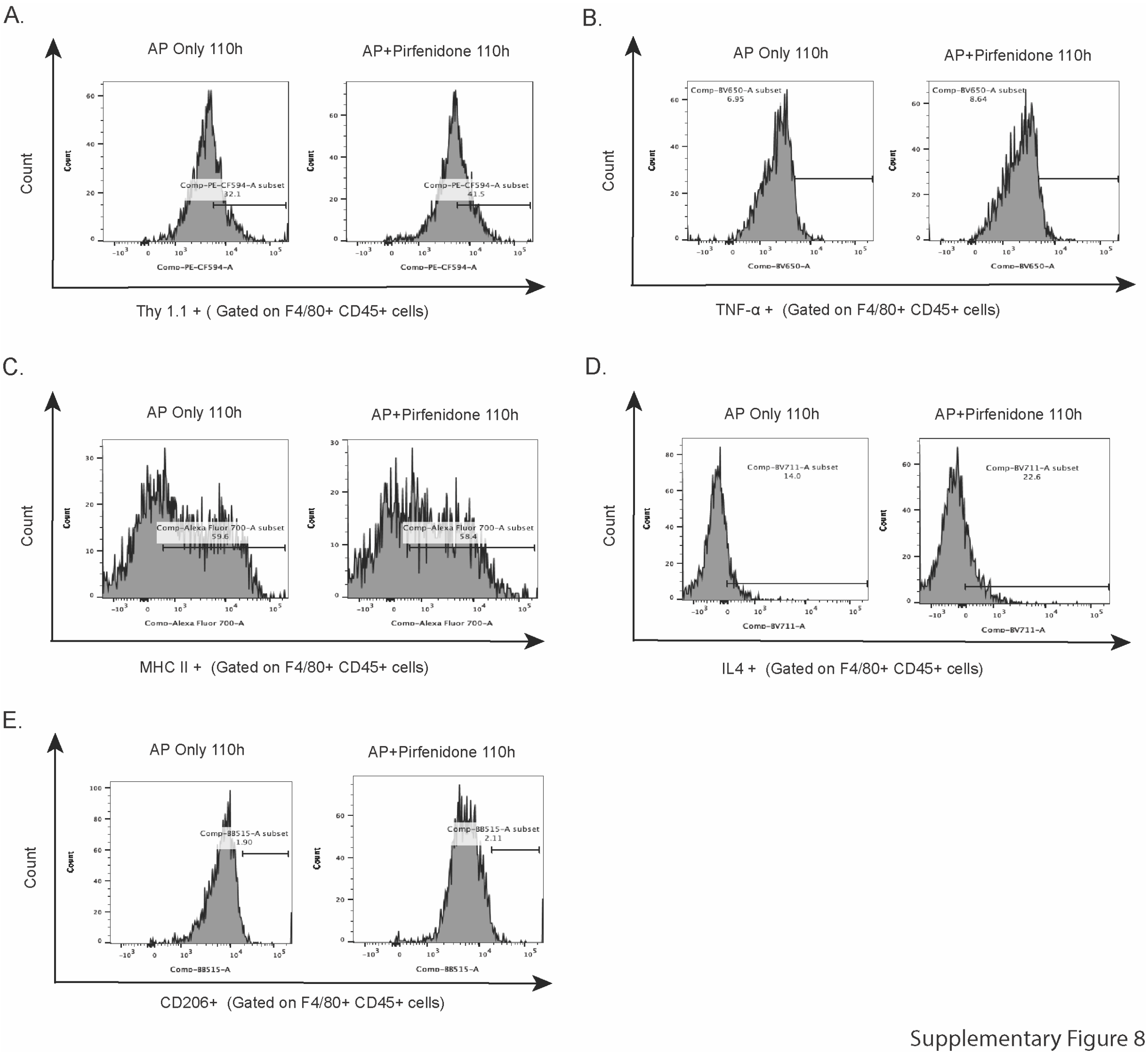

Supplementary Figure 8:

(A-E) Gating strategy for flow cytometry analysis of pancreatic immune cells at 110h time point in IL10 reporter mice.

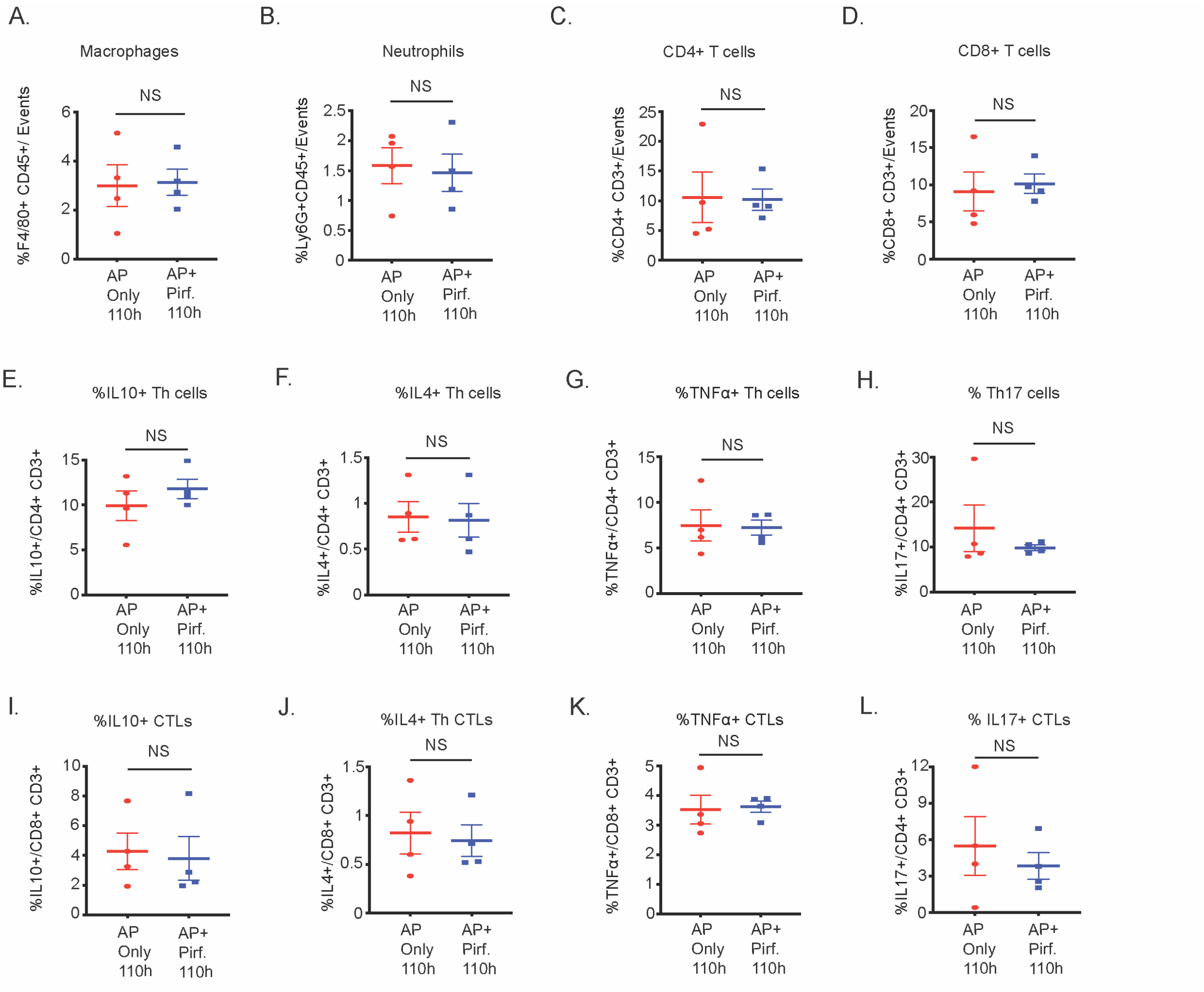

Supplementary Figure 9

Supplementary Figure 9:

(A-L) Flow cytometry analysis for macrophage/neutrophil and T cell panel at 110h time point of L-arginine AP which did not show a statistically significant difference with pirfenidone treatment. Pirf.=Pirfenidone; NS=Not significant. Data represents mean± SEM and n =4 in each group. *P < .05 (Mann Whitney test).

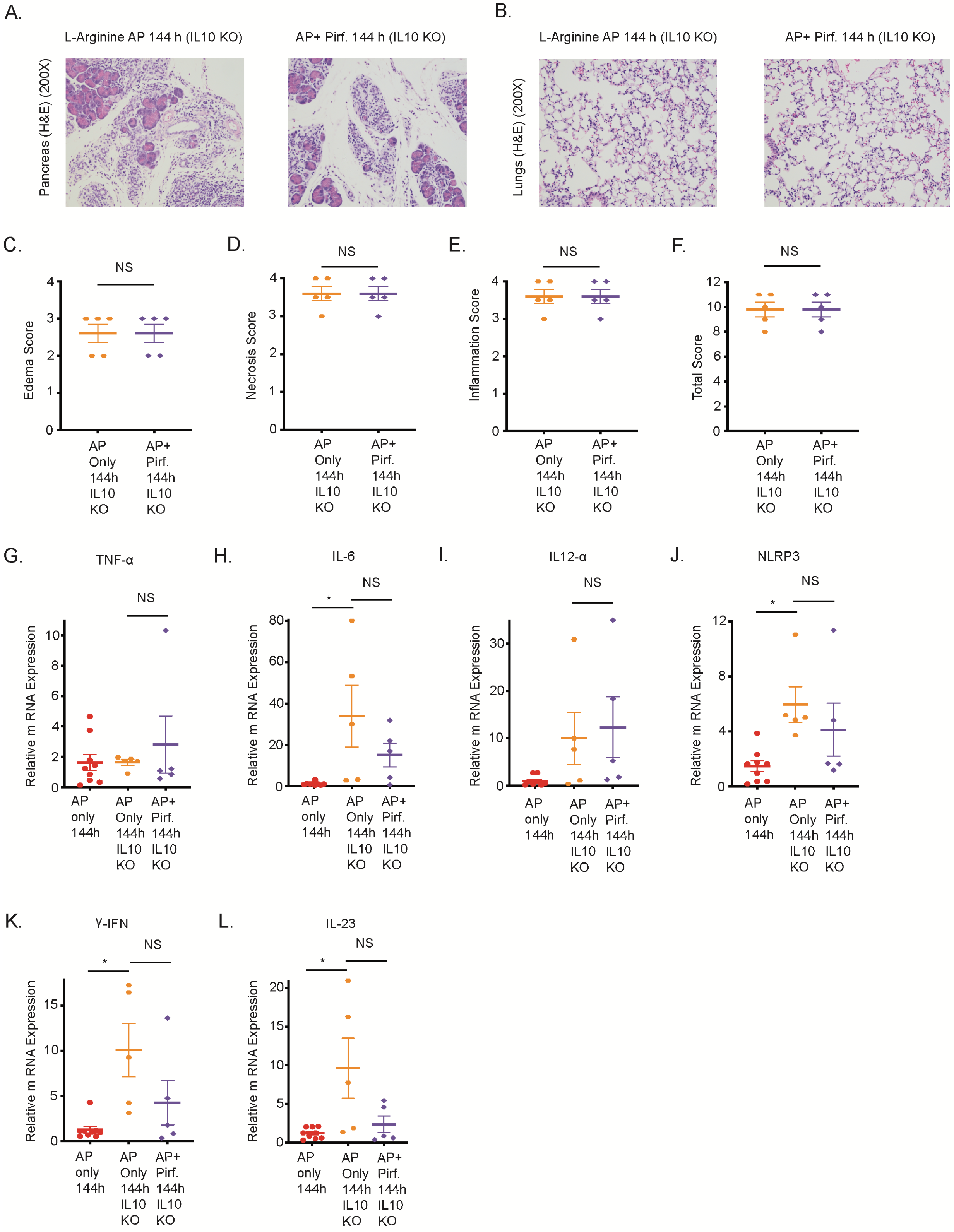

Supplementary Figure 10

Supplementary Figure 10: Therapeutic pirfenidone, when administered at the peak of injury in a well-established L-arginine model of acute pancreatitis in IL10KO mice (C57Bl/6 background), does not ameliorate local pancreatic and lung injury at 144h.

(A) Histological analysis of representative H&E sections (200X) of pancreas from AP only group and pirfenidone treated AP group shows no improvement with pirf. in IL10KO mice. (B) Lung H&E (200X) at 110h does not show a reduction in injury with pirfenidone treatment in IL10KO mice. (C-F) Histology scoring shows no difference in pancreatic edema, necrosis and inflammatory infiltrate in IL10KO mice with pirf. treatment. (G-L) mRNA levels of proinflammatory markers TNF-α, IL-6, IL-12α, NLRP3, IFN-γ and IL-23 did not show a significant decrease with pirfenidone treatment in IL10KO mice. Pirf.=Pirfenidone; NS=Not significant. n =5 in each group. Data represents mean± SEM and *P < .05 [Kruskal-Wallis Test (Dunn's Multiple Comparisons Test)].

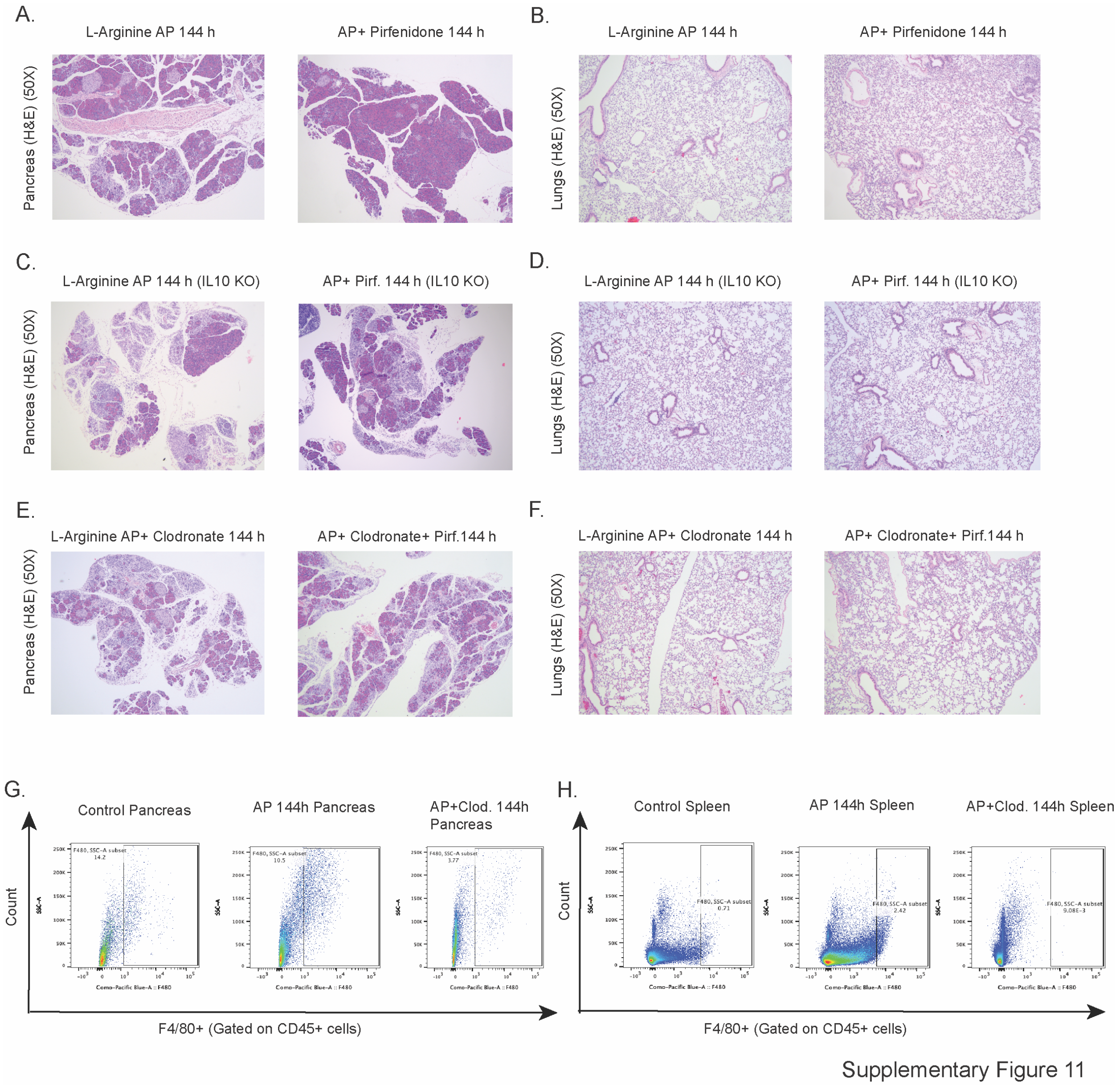

Supplementary Figure 11 :

(A-F) Histological analysis of representative H&E sections (50X) of pancreas and lung respectively, from AP only group and pirfenidone treated AP group, in wild type C57Bl/6 (A-B), IL10KO on C57Bl/6 background(C-D) and C57Bl/6 with clodronate liposome induced depletion of macrophages (E-F). (G-H) Gating strategy for confirmation of macrophage depletion using clodronate in pancreas (G) and spleen (H). Pirf.=Pirfenidone.
